# Reference values for wrist-worn accelerometer physical activity metrics in England children and adolescents

**DOI:** 10.1186/s12966-023-01435-z

**Published:** 2023-03-25

**Authors:** Stuart J. Fairclough, Alex V. Rowlands, Borja del Pozo Cruz, Matteo Crotti, Lawrence Foweather, Lee E. F. Graves, Liezel Hurter, Owen Jones, Mhairi MacDonald, Deborah A. McCann, Caitlin Miller, Robert J. Noonan, Michael B. Owen, James R. Rudd, Sarah L. Taylor, Richard Tyler, Lynne M. Boddy

**Affiliations:** 1 Movement Behaviours, Nutrition, Health, & Wellbeing Research Group, and Department of Sport & Physical Activity, Edge Hill University, Ormskirk, UK; 2 Diabetes Research Centre, Leicester General Hospital, University of Leicester, Leicester, UK; 3 National Institute for Health Research (NIHR) Leicester Biomedical Research Centre (BRC), University Hospitals of Leicester NHS Trust and the University of Leicester, Leicester, UK; 4 Faculty of Education, University of Cádiz, Cádiz, Spain; 5 Biomedical Research and Innovation Institute of Cádiz (IMiBICA) Resarch Unit, Puerta del Mar University Hospital, University of Cádiz, Cádiz, Spain; 6 Department of Sports Science and Clinical Biomechanics, Centre for Active and Healthy Ageing, University of Southern Denmark, Odense, Denmark; 7 Research Centre for Sport, Exercise, and Life Sciences, Coventry University, Coventry, UK; 8 The Physical Activity Exchange, Research Institute of Sport and Exercise Sciences, Liverpool John Moores University, Liverpool, UK; 9 Faculty of Health and Wellbeing, University of Bolton, Bolton, UK; 10 Department of Applied Health and Social Care and Social Work, Faculty of Health, Social Care and Medicine, Edge Hill University, Ormskirk, UK; 11 Norwegian School of Sport Sciences, Oslo, Norway

**Keywords:** ENMO, MAD, Youth, Raw data, Average acceleration, Intensity gradient, MX metrics, MVPA

## Abstract

**Background:**

Over the last decade use of raw acceleration metrics to assess physical activity has increased. Metrics such as Euclidean Norm Minus One (ENMO), and Mean Amplitude Deviation (MAD) can be used to generate metrics which describe physical activity volume (average acceleration), intensity distribution (intensity gradient), and intensity of the most active periods (MX metrics) of the day. Presently, relatively little comparative data for these metrics exists in youth. To address this need, this study presents age- and sex-specific reference percentile values in England youth and compares physical activity volume and intensity profiles by age and sex.

**Methods:**

Wrist-worn accelerometer data from 10 studies involving youth aged 5 to 15 y were pooled. Weekday and weekend waking hours were first calculated for youth in school Years (Y) 1&2, Y4&5, Y6&7, and Y8&9 to determine waking hours durations by age-groups and day types. A valid waking hours day was defined as accelerometer wear for ≥ 600 min·d^−1^ and participants with ≥ 3 valid weekdays and ≥ 1 valid weekend day were included. Mean ENMO- and MAD-generated average acceleration, intensity gradient, and MX metrics were calculated and summarised as weighted week averages. Sex-specific smoothed percentile curves were generated for each metric using Generalized Additive Models for Location Scale and Shape. Linear mixed models examined age and sex differences.

**Results:**

The analytical sample included 1250 participants. Physical activity peaked between ages 6.5–10.5 y, depending on metric. For all metrics the highest activity levels occurred in less active participants (3^rd^-50^th^ percentile) and girls, 0.5 to 1.5 y earlier than more active peers, and boys, respectively. Irrespective of metric, boys were more active than girls (*p* < .001) and physical activity was lowest in the Y8&9 group, particularly when compared to the Y1&2 group (*p* < .001).

**Conclusions:**

Percentile reference values for average acceleration, intensity gradient, and MX metrics have utility in describing age- and sex-specific values for physical activity volume and intensity in youth. There is a need to generate nationally-representative wrist-acceleration population-referenced norms for these metrics to further facilitate health-related physical activity research and promotion.

**Supplementary Information:**

The online version contains supplementary material available at 10.1186/s12966-023-01435-z.

## Background

Accelerometers are commonly used in physical activity and health research concerning surveillance [1, 2], epidemiology [3, 4], and intervention evaluation [5, 6]. The utility of accelerometers is well established and evidenced by observed relationships between physical activity and health outcomes across the life-course including all-cause mortality [3], cardiovascular disease [7], cancer [8], obesity [9], musculoskeletal health [10], and psychosocial wellbeing [11].

Historically, most accelerometer data have been derived from hip-worn devices, but over the last decade there has been a shift towards alternative wear sites, such as the wrist, thigh, and back [2, 12, 13] to improve participant compliance to wear protocols and therefore enhance the reliability of resultant data [14, 15]. In particular, large scale cohort studies such as the UK Biobank [2] and the Brazilian Birth Cohort Study [16] have employed wrist-worn devices to produce population-level data associated with health. Similarly, the US NHANES and the NHANES National Youth Fitness Survey moved from hip- to wrist-worn physical activity assessments to reduce participant burden, resulting in an increase in device wear compliance [17–19]. This change also provided a more secure and comfortable accelerometer attachment site which aligns better with the 24-h movement behaviours paradigm by capturing data over the 24-h cycle [20].

In step with these changes has been the increased transition from proprietary accelerometer metrics (i.e., counts), to use of potentially device-agnostic raw acceleration data-driven metrics. Metrics such as Euclidean Norm Minus One (ENMO) [21], Mean Amplitude Deviation (MAD) [22], and Monitor Independent Movement Summary (MIMS) units [23] provide composite summary acceleration values and have been increasingly used over the last decade. These metrics can be generated in a relatively straightforward and cost-effective manner due to the increased accessibility of raw acceleration data and open-source processing and analysis applications, such as the GGIR and MIMS-unit R packages [24, 25].

The potential utility of such metrics was recently expanded by Belcher et al. who published MIMS-units US population-referenced percentiles for wrist-worn accelerometry [1]. This study, which was based on similar analyses of hip-worn ActiGraph counts from 2014 [26] and 2015 [27], included children and adolescents aged 3- to 19-years and adults up to age 80 + years. The authors reported MIMS-units in over 6000 youth and found that activity peaked in both sexes at age 6 years and were lowest at age 17 years and 18 years in males and females, respectively. It was also observed that females accumulated higher MIMS-units than males at lower percentiles (< 50^th^), while the opposite was observed at the higher percentiles up to age 11 years [1]. The analysis of Belcher and colleagues provides unique insights into age- and sex-related activity differences derived from wrist raw acceleration metrics, which in some instances were counter to those previously reported for hip-worn accelerometer proprietary counts data (e.g., males being more active than females at all ages) [1].

In addition to MIMS-units, ENMO and, to a lesser extent, MAD metrics have been used as summary acceleration metrics with increasing frequency. Presently, the differences and patterns in youth physical activity for both summary metrics based on average acceleration (i.e., proxy for activity volume) [28] are unknown. Furthermore, age- and sex-related differences have not been described for additional device-agnostic metrics that describe the specific physical activity dimensions of intensity (i.e., intensity gradient) [28], and time-related intensity (i.e., MX: minimum acceleration for the most active accumulated period of time, where X = the period of time) [29]. Average acceleration and intensity gradient are independently-related to various health and wellbeing outcomes in different populations [28, 30, 31], while MX metrics can be used to estimate prevalence of meeting physical activity guidelines [29, 32]. As the use of these metrics continues to increase, there is a need for reference values to help the physical activity and health research community interpret activity levels from continuous raw acceleration data [33]. However, relatively little comparative data for average acceleration, intensity gradient, and MX metrics are available for children and adolescents, and where it does exist it is limited by narrow age groups and/or modest sample sizes [28, 34, 35]. To address this need our aims for this novel study were:

1. To present age- and sex-group reference percentile values for ENMO- and MAD-generated average acceleration, intensity gradient, and MX metrics in a wide age-range of England children and adolescents;
2. To compare volume and intensity physical activity profiles for ENMO- and MAD-generated metrics by age- and sex-groups.

## Methods

### Data acquisition and study eligibility

Ten ethically approved wrist accelerometry studies led or supervised by the first or last authors were identified for inclusion in this pooled individual participant data analysis. Eligible studies involved school-aged youth who provided assent and who had parental/carer written informed consent to participate in physical activity research studies during school term time. Seven studies were cross-sectional and three were interventions, six focused on primary school students only, two focused on secondary school students, and two studies included primary and secondary school students. Participant inclusion criteria varied by study but as a minimum, participants were required to be physically able to regularly take part in physical education classes. Individual study sample sizes ranged from *n* = 29 to 311, with a mean of *n* = 150 ± 84 participants. Seven studies included recruitment information to determine the participation rate (mean = 72%). The participants’ socio-economic position (SEP) spanned English Indices of Multiple Deprivation deciles 1 (low SEP) through 10 (high SEP) [36]. The median decile was 4 (IQR = 2, 8) which reflects the established health inequalities in northwest England. Participant characteristics are presented by study in Additional file 14. For inclusion in the analysis, studies required non-intervention assessments of wrist accelerometer-derived physical activity. For the included intervention studies only baseline data were used. In addition to raw acceleration data, as a minimum, studies needed to provide stature, body mass, and demographic data including age and sex. Where published, details of these studies can be found elsewhere [37–42]. Investigators with a major involvement in the eligible studies (e.g., past PhD students, co-supervisors) were approached by email and invited to contribute individual participant data to allow data harmonisation and subsequent pooled analysis. On receipt of signed data transfer agreements all contributing investigators transferred their de-identified data via a secure file sharing system. Ethical approval for this pooled individual participant data study was granted by Edge Hill University’s Science Research Ethics Committee (#ETH2021-0034). Data were available from 10 studies conducted in 71 schools between 2015 and 2022 in the Merseyside, Lancashire, and Greater Manchester counties of northwest England.

### Anthropometric and demographic variables

In all contributing studies stature and body mass were measured to the nearest 0.1 cm/kg using a portable stadiometer and digital scales, respectively. Standard anthropometrical procedures were followed with participants wearing light clothing and shoes removed [43]. International Obesity Task Force age- and sex-specific body mass index (BMI) cut-points were applied to classify participants by weight status [44], and BMI z-scores were computed using UK 1990 reference data [45].

### Physical activity acceleration metrics

In the contributing studies ActiGraph GT9X (ActiGraph, Pensacola, FL; 8 studies), or GENEActiv Original (Activinsights, Cambs, UK; 2 studies) triaxial accelerometers were used. The devices have a dynamic range of ± 8 g and were requested to be worn for up to 7 consecutive days on the non-dominant wrist using either 24-h (8 studies) or waking hours wear protocols (2 studies), with a sampling frequency set at 100 Hz (8 studies) or 30 Hz (2 studies). The devices were initialised and data downloaded using the latest releases of the respective ActiLife (versions 6.13.1 to 6.13.4) and GENEActiv (versions 2.2 to 3.1) available at the time of data collection. Physical activity metrics were generated from the raw accelerometer data files (ActiGraph: gt3x then conversion to.csv format; GENEActiv:.bin format) and were processed in R using package GGIR version 2.6–0 [24].

#### Accelerometer data harmonisation

To harmonise data collected from 24-h and waking hours protocols it was first necessary to define the age and day-specific waking windows of interest, as follows: Firstly, accelerometer files were sorted into four age-groups based on school Year (i.e., Grade) group (Year (Y) 1&2 (age 5–7 y), Y4&5 (age 8–10 y), Y6&7 (age 10–12 y), and Y8&9 (age 12–14 y), which were used in all subsequent steps. Next, accelerometer files from 24-h data collection protocol studies were processed in GGIR parts 1 to 4 using the default sleep detection algorithm [46] to estimate average waking and sleep times (and therefore duration of a ‘waking hours’ day) for weekdays and weekend days. Accelerometer files for 1297 participants were processed with the resultant sleep data representing 5627 participant-days. The averaged waking and sleep times, and total awake duration for each group and day type (i.e., weekdays, weekend days) are presented in Table 1.

**Table 1:** Weekday and weekend averaged wake and sleep times (Mean (SD)) and waking hours duration

	Weekday			Weekend		
Age groups	Waking time	Sleep time	Total awake duration (min)	Waking time	Sleep time	Total awake duration (min)
Y1&2	07:20 (00:16)	21:16 (00:21)	837	07:59 (00:33)	21:49 (00:29)	830
Y4&5	07:04 (00:11)	21:35 (00:22)	871	07:43 (00:35)	21:55 (00:49)	852
Y6&7	07:14 (00:28)	22:16 (00:21)	902	08:05 (00:35)	22:42 (00:35)	867
Y8&9	07:09 (00:50)	22:49 (00:27)	940	08:02 (01:21)	23:22 (00:41)	860

*Note*: Times are in 24-h clock format

These averaged waking and sleep times were then used in separate GGIR shell R scripts to populate the *qwindows* argument to define waking hours during subsequent data processing. This ensured that determination of the waking day for data processing was specific for each age group and day type. Age group accelerometer files, including those from the two studies that did not use a 24-h wear protocol, were then re-processed separately in GGIR part 2 to calculate the ENMO and MAD-derived waking hours physical activity acceleration outcome metrics. This processing was undertaken for weekdays and for weekend days for each of the four age groups (i.e., eight separate data processing runs). Signal processing included autocalibration using local gravity as a reference [47], detection of implausible values, and detection of non-wear. Non-wear was imputed by default in GGIR whereby invalid data were imputed by the average at similar time points on other days of the week [21]. An example GGIR configuration file contains details of the parameters selected (Additional file 1). Wear time criteria were: at least three valid weekdays and one valid weekend day, with ≥ 600 min·d^–1^ of accelerometer wear during waking hours defined as a valid wear day. Participants’ accelerometer data were excluded from analyses if post-calibration error was > 10 mg (milli-gravitational units) and/or the wear time criteria were not achieved.

#### Acceleration metrics

Average acceleration (i.e., average magnitude of dynamic acceleration) was calculated during GGIR part 1 processing using ENMO (i.e., the Euclidean norm of the three accelerometer axes with 1 g subtracted and negative values truncated to zero [21], and MAD (i.e., the mean of the dynamic acceleration signal with the static component removed) [48]. To reflect the intermittent nature of youth physical activity behaviour and to ensure higher intensity activities were captured both summary metrics were averaged over 1-s epochs [49, 50]. The metrics were expressed in m*g* to represent activity volume and were used to generate all subsequent metrics. Average acceleration from the ActiGraph and GENEActiv devices worn on the non-dominant wrist has demonstrated equivalence in adults [51].

The intensity gradient reflects the negative curvilinear relationship between intensity and time accumulated at any given intensity, and describes an individual’s intensity profile during the measurement period [28]. A higher intensity gradient (i.e., less negative value) reflects proportionately more time being spread across the intensity profile, whereas a lower or more negative gradient reflects proportionately less time spent in mid-range and higher intensities. Intensity gradient was selected in GGIR part 2 using the *iglevels* = *TRUE* argument.

The MX metric (where X refers to an accumulated duration of time) is the acceleration in m*g* above which the most active X minutes are accumulated. The MX metric is a population-independent continuous variable, derived from directly measured acceleration, and captures intensity irrespective of level of activity, or fitness status (unlike absolute intensity cut-points) [29]. Fourteen different MX metrics were computed to cover different durations of interest and thus give a comprehensive picture of profile of physical activity. These were M2, M5, M10, M15, M20, M30, M45, M60, M120, M240, M360, M480, M600, and M720. These metrics were generated in GGIR part 2 using the *qlevels* argument aligned to the waking hours duration indicated by the *qwindows* time range.

To allow comparisons with previous studies employing wrist-accelerometer cut-points, we also calculated time spent in moderate-to-vigorous physical activity (MVPA), using an ENMO threshold of 200 mg, which approximates the ActiGraph and GENEActiv cut-points of 201.4 mg and 191.6 mg, respectively [52]. Using 200 mg allowed comparison with previous studies that had used either device [28, 31]. We did not calculate MVPA for MAD because to our knowledge published cut-points for non-dominant wrist data in youth do not exist for this summary metric. Additional file 2 provides a summary of the GGIR output variables selected.

#### Data analysis

Processed accelerometer data for each age group were firstly combined and then average weighted week (5:2 ratio) values were computed for average acceleration, intensity gradient, MX metrics, and MVPA (ENMO only). These data were then harmonised with the corresponding anthropometric and demographic data using each participant’s unique ID code. Sex- and age-group descriptive statistics were calculated for average acceleration, intensity gradient, M60 (which relates most closely to the youth physical activity guideline of at least 60 min MVPA·d^−1^ averaged across the week), and MVPA. We used the gamlss R package (v.5.4–10) [53] to create sex- and age-specific percentile curves (3^rd^, 5^th^, 10^th^, 25^th^, 50^th^, 75^th^, 90^th^, 95^th^, and 97^th^) for each metric using the Generalized Additive Models for Location Scale and Shape (GAMLSS) method [54]. All metrics were modelled at each age (in increments of 0.1 years) with a parametric distribution (Box-Cox t, Box-Cox Power Exponential, or Box-Cox normal; depending on which distribution had the best model fit). The characteristics of the chosen parametric distribution (i.e., the location, scale, skewness, and kurtosis) were then modelled to vary smoothly across age using penalised B-splines [55]. Goodness of fit was checked for all models using worm plots.

The GAMLj R package (v. 2.4.0) [56] was used to generate linear-mixed models to examine age and sex differences for each metric, while accounting for individual participant data being clustered in schools. Season of data collection, accelerometer wear time, accelerometer type, and recording frequency were included as covariates. Radar plots were constructed to present the sex- and age-group MX metrics. These provide a visual translation of between-group activity intensity profiles. Each MX metric is plotted on one radius and the points joined to give a distinct shape for each age- and sex-group. Higher intensity profiles are indicated by a greater surface area of the plotted shape on the left of the plot, where the shorter duration MX metrics were positioned (i.e., M30 through to M2) [33]. To enable translation of the MX metrics with traditional time-use intensity thresholds, dashed lines are included in the plots at 200 mg and 700 mg, to represent moderate (MPA) and vigorous physical activity (VPA), respectively [52]. To reflect that ENMO has been used more extensively than MAD in a range of age groups and populations [28, 30–32], we report our results on the former. Equivalent data for MAD are presented as supplementary material (Additional files 3, 4, 5, 6, 7 and 8)

## Results

**Fig. 1:**
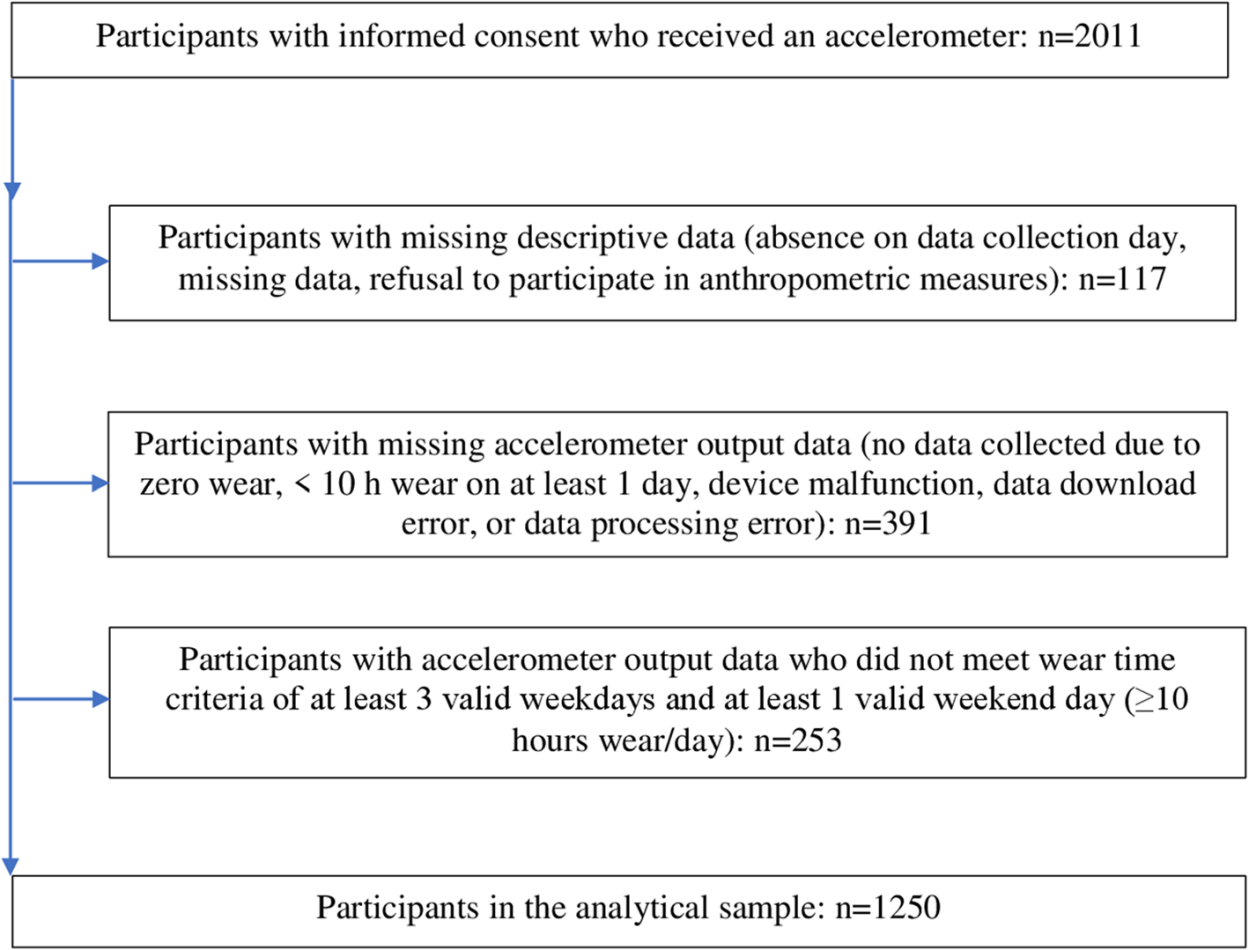
Data flowchart for the analytical sample

From the 10 contributing studies *n* = 2011 participants had informed parental consent to participate. When participants with missing descriptive data and without accelerometer output data were removed the sample was *n* = 1503 (75% of consented sample). Two-hundred-and-fifty-three participants did not achieve the accelerometer wear time criteria, resulting in a final analytical sample of *n* = 1250 (62.2% of consented sample and 83.2% of sample with accelerometer data; Fig. 1).

There were no significant differences in age (*p* = 0.26), BMI z-score (*p* = 0.98) or sex (*p* = 0.09) between the participants that achieved wear time compliance and those that did not. Participant descriptive characteristics for the analytical sample are presented in Table 2. The sample consisted of 59% girls, which was mainly due to there being substantially more girls than boys in the 12–14 y age group. This reflected the inclusion of a girls-only study in the pooled dataset. The proportion of participants classified as normal-weight ranged from 69 to 77% (boys) and from 70 to 78% (girls), which is broadly in line with national data in England corresponding with the years when the included studies were conducted [57]. Compliance to the accelerometer wear protocol was very good among participants in the analytical sample (Table 3). On average the accelerometers were worn for 6.0 days out of 7 for 14.0 h·d^−1^ with the highest compliance seen in the older groups, and lowest compliance in the youngest group.

**Table 2:** Participants’ descriptive characteristics, grouped by sex and age (Mean (SD), unless stated otherwise)

	Boys				Girls			
	Y1&2	Y4&5	Y6&7	Y8&9	Y1&2	Y4&5	Y6&7	Y8&9
*n*	82	201	163	64	95	253	155	237
Age (y)	6.0 (0.3)	9.7 (0.5)	10.7 (0.6)	13.0 (0.4)	6.0 (0.3)	9.7 (0.5)	10.7 (0.7)	13.7 (0.5)
Height (cm)	116.5 (6.2)	138.3 (6.7)	143.8 (8.0)	160.0 (8.8)	115.8 (5.1)	138.2 (7.2)	144.3 (7.7)	160.2 (6.4)
Weight (kg)	22.3 (3.7)	34.5 (8.5)	38.8 (10.1)	53.5 (11.7)	22.0 (3.7)	35.7 (8.4)	40.3 (11.0)	55.2 (11.3)
BMI (kg**‧**m^2^)	16.3 (1.7)	17.9 (3.1)	18.6 (3.5)	20.8 (4.1)	16.3 (2.1)	18.5 (3.2)	19.2 (4.0)	21.4 (3.9)
BMI-z	0.39 (0.97)	0.48 (1.20)	0.46 (1.38)	0.79 (1.15)	0.38 (1.30)	0.55 (1.12)	0.47 (1.31)	0.58 (1.17)
Normal-weight (%)	73.0	77.0	72.0	69.0	78.0	70.0	72.0	76.0
Overweight/obese (%)	27.0	23.0	28.0	31.0	22.0	30.0	28.0	24.0

**Table 3:** Participants’ unadjusted waking hours average weighted week accelerometer data, grouped by sex and age (Mean (SD), unless stated otherwise)

	Boys				Girls			
	Y1&2	Y4&5	Y6&7	Y8&9	Y1&2	Y4&5	Y6&7	Y8&9
*n*	82	201	163	64	95	253	155	237
Valid days (n)	5.1 (0.5)	6.4 (0.7)	6.2 (0.7)	6.1 (0.6)	5.1 (0.4)	6.4 (0.7)	6.3 (0.6)	6.6 (0.7)
Wear (h**‧**d^−1^)	13.6 (0.8)	13.9 (0.8)	14.3 (0.8)	14.5 (0.8)	13.6 (0.6)	13.9 (0.8)	14.4 (0.7)	14.6 (0.8)
Average acceleration (m*g)*	75.9 (19.8)	73.4 (21.5)	74.0 (25.5)	59.2 (27.1)	65.1 (13.6)	62.1 (17.3)	64.3 (17.1)	43.6 (12.2)
Intensity gradient	-2.07 (0.12)	-2.04 (0.12)	-2.07 (0.14)	-2.20 (0.18)	-2.15 (0.11)	-2.14 (0.12)	-2.16 (0.14)	-2.38 (0.17)
M2 (m*g*)	1949.1 (423.2)	2088.8 (530.2)	2018.1 (607.1)	1445.1 (662.5)	1552.5 (345.3)	1664.3 (439.7)	1661.0 (519.4)	899.8 (392.0)
M5 (m*g*)	1386.2 (319.0)	1425.1 (428.6)	1377.5 (481.8)	936.7 (520.9)	1081.3 (262.7)	1096.1 (328.6)	1095.6 (369.8)	593.5 (277.1)
M10 (m*g*)	963.7 (240.8)	960.4 (323.5)	937.9 (362.6)	634.4 (381.6)	744.4 (188.9)	723.6 (230.9)	733.3 (249.0)	417.1 (210.3)
M15 (m*g*)	737.01 (198.3)	726.5 (258.9)	716.1 (289.0)	501.0 (325.1)	570.8 (142.5)	547.3 (175.0)	560.9 (183.4)	341.1 (191.0)
M20 (m*g*)	593.0 (165.7)	583.4 (213.4)	580.6 (237.9)	421.2 (283.1)	463.9 (111.5)	444.8 (139.2)	458.5 (143.0)	291.6 (165.7)
M30 (m*g*)	423.4 (121.1)	417.1 (146.4)	423.6 (170.2)	324.1 (205.1)	339.6 (73.8)	329.7 (97.1)	343.0 (97.9)	236.3 (149.7)
M45 (m*g*)	287.4 (82.9)	295.0 (95.9)	303.4 (112.4)	246.3 (128.9)	246.9 (47.9)	244.2 (66.7)	256.3 (66.3)	183.84 (45.8)
M60 (m*g*)	230.8 (61.4)	230.2 (69.0)	238.3 (82.0)	204.6 (109.4)	196.7 (35.8)	196.9 (51.5)	207.8 (50.5)	154.9 (33.4)
M120 (m*g*)	122.1 (30.5)	121.5 (31.7)	129.07 (40.9)	112.9 (33.0	108.8 (19.6)	110.6 (28.7)	119.5 (27.7)	93.8 (21.9)
M240 (m*g*)	52.9 (13.7)	51.7 (14.3)	57.4 (19.9)	49.7 (17.6)	48.6 (10.7)	49.3 (15.1)	54.2 (14.7)	41.6 (12.0)
M360 (m*g*)	41.8 (10.9)	38.6 (10.8)	41.1 (14.5)	33.5 (12.6)	38.8 (8.9)	37.2 (11.95)	38.7 (10.9)	28.3 (8.6)
M480 (m*g*)	26.8 (6.8)	26.3 (7.4)	29.6 (10.4)	24.8 (9.1)	25.7 (6.1)	25.7 (8.5)	27.6 (7.9)	21.4 (6.7)
M600 (m*g*)	14.8 (4.0)	14.4 (4.5)	16.5 (6.1)	13.6 (5.2)	14.5 (4.1)	14.2 (5.4)	15.1 (4.7)	12.0 (4.3)
M720 (m*g*)	7.0 (2.7)	7.0 (3.0)	8.7 (3.9)	7.3 (3.4)	7.1 (2.7)	7.0 (3.5)	7.8 (3.0)	6.2 (2.8)
MVPA (min**‧**d^−1^)	67.1 (21.1)	65.8 (22.1)	68.2 (27.7)	53.9 (27.2)	56.5 (15.0)	55.2 (20.3)	59.8 (20.8)	36.5 (15.0)

*Notes*. Accelerometer outcomes calculated using the ENMO metric; MX metrics = minimum acceleration for the most active accumulated X minutes; MVPA = Moderate-to-vigorous physical activity

### Results for study aim 1

**Fig. 2:**
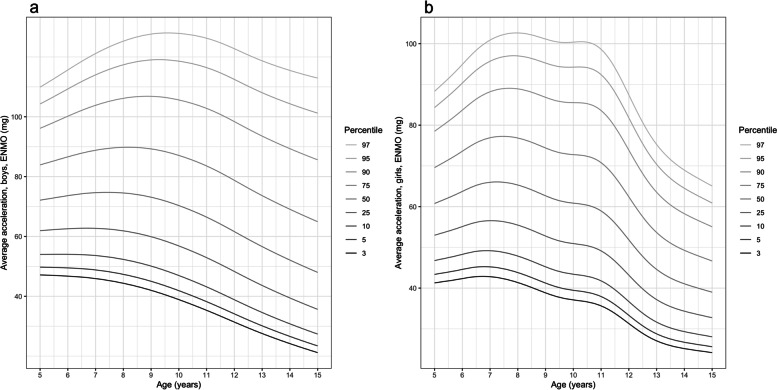
Percentiles of waking hours wrist-worn average acceleration for boys (panel **a**) and girls (panel **b**)

Figure 2 displays the age-specific average acceleration percentiles for boys and girls. At all ages boys’ average acceleration was greater than girls across the full percentile range. Average acceleration peaked between ages 6.5 and 7.5 y in the less active boys (up to 50^th^ percentile) and between 8.5 and 9.5 y in the more active boys, compared to ages 7 and 7.5 y in the less active girls (up to 50^th^ percentile) and 7.5 and 8 y in the more active girls. The observed age-related decline was gradual in boys and relatively steep in girls from age 11 y. Girls’ average acceleration declined most from age 11y with the steepest reductions in the most active girls (i.e., 50^th^-97^th^ percentile).

**Fig. 3:**
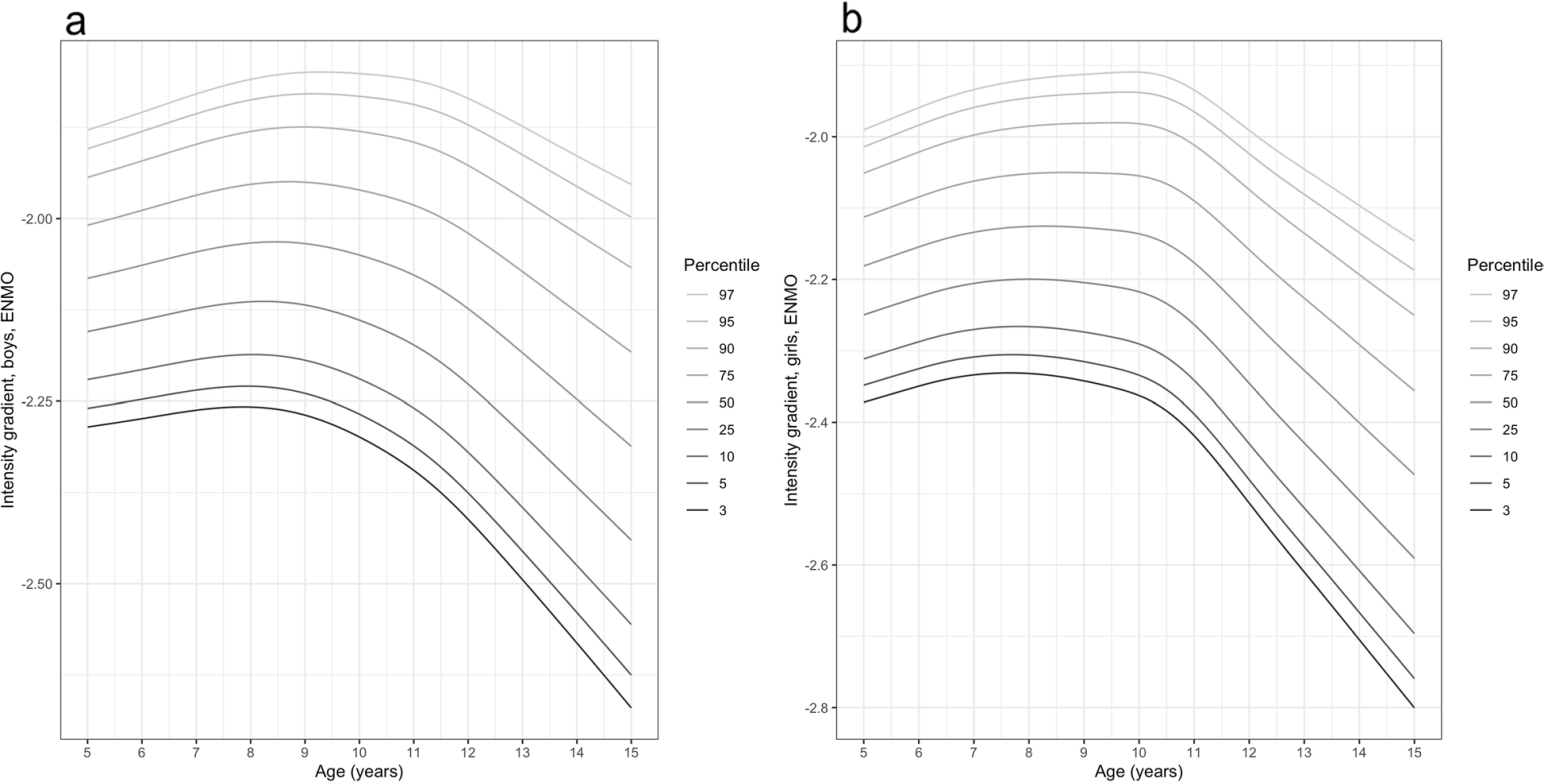
Percentiles of waking hours wrist-worn intensity gradient for boys (panel **a**) and girls (panel **b**)

Figure 3 presents age- and sex-specific percentiles for intensity gradient. At all ages and activity levels boys’ intensity gradient values were higher (less negative) than girls. Intensity gradient was highest between ages 8–9 y and between 7.5–8.5 y in the less active boys and girls, respectively (up to 50^th^ percentile). Among the most active, intensity gradient peaked between 9.5 and 10 y (girls) and between 9 and 10 y (boys). In both sexes the age-related decline in intensity gradient was somewhat steeper than for average acceleration and was most pronounced from age 10 to 11.5 y, with the less active participants reducing intensity gradient earlier than more active peers.

**Fig. 4:**
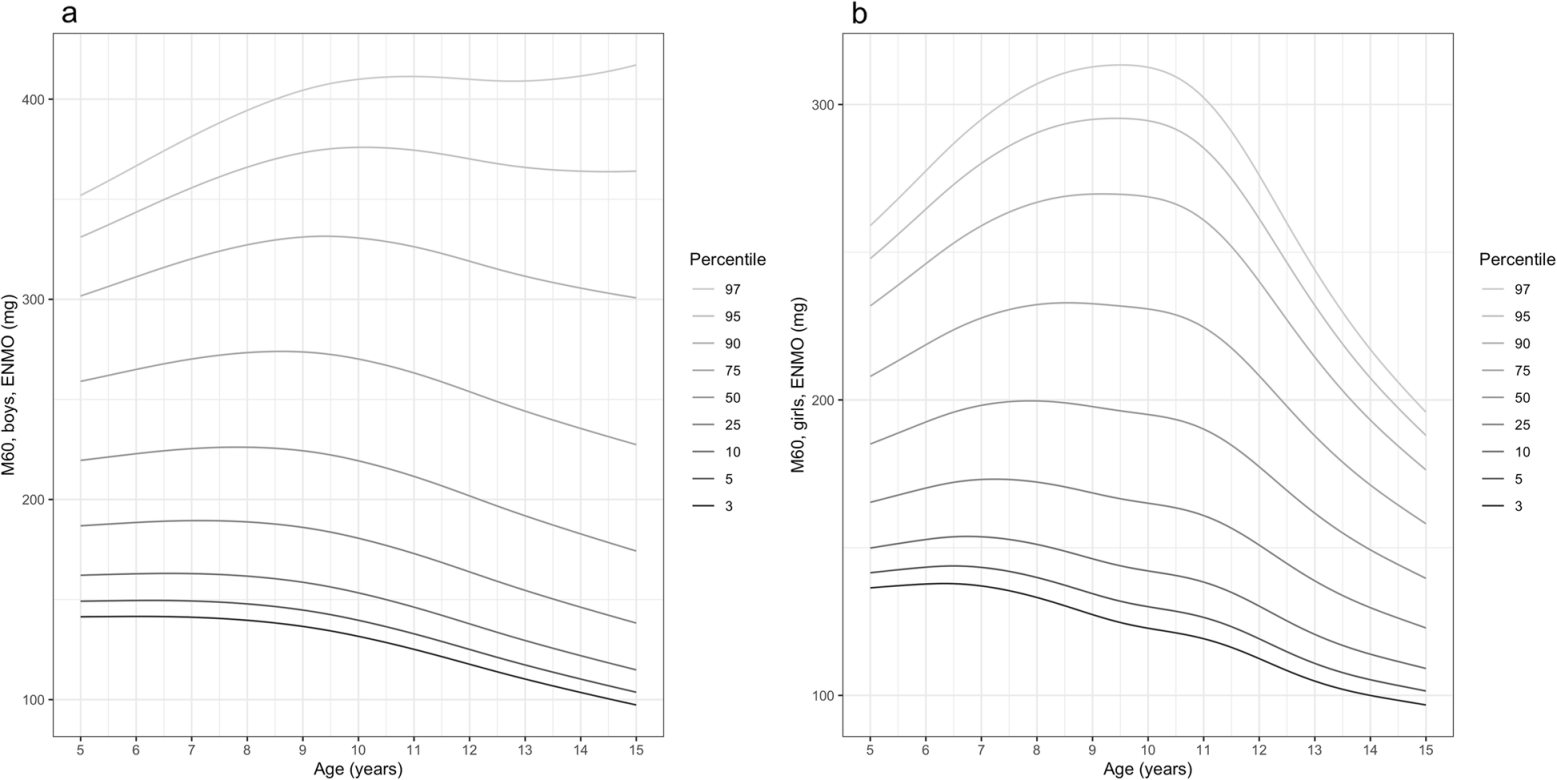
Percentiles of waking hours wrist-worn M60 metric for boys (panel **a**) and girls (panel **b**)

Age- and sex-specific M60 percentiles are shown in Fig. 4, with girls’ values consistently lower than boys at all ages. M60 was highest between ages 7.5–8.5 y in the less active boys (up to 50^th^ percentile) and between 9.5 and 11 y in the more active boys. In girls M60 peaked between ages 6.5–8 y (up to 50^th^ percentile) and between 8.5 and 9.5 y (50^th^ -97^th^ percentile). M60 declined gradually in boys across almost all percentiles, although at age 13–14 y the slope plateaued in the most active group (97^th^ percentile). However, at the extremes of the curves only a small number of participants were represented (e.g., 15 boys in the 97% percentile), therefore these data should be interpreted with caution. In contrast, girls’ M60 values fell most from age 11 y with the steepest reductions at the 50^th^-97^th^ percentiles.

**Fig. 5:**
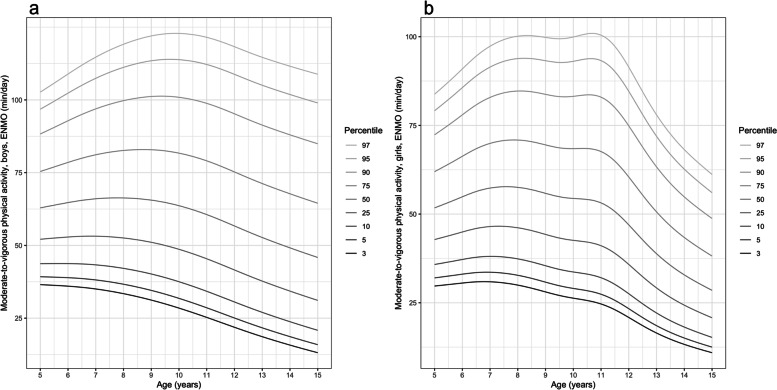
Percentiles of waking hours wrist-worn MVPA for boys (panel **a**) and girls (panel **b**)

Figure 5 displays time spent in MVPA. At all ages boys’ MVPA was greater than girls’ at each percentile. Among the less active participants (up to 50^th^ percentile) MVPA peaked between ages 6.5–8.5 y in the less active boys and between 8.5 and 9.5 y in the more active boys. In more active peers MVPA was highest between 8.5 and 9.5 y (boys) and between 8 and 10.5 y (girls). The age-related decline in MVPA was gradual in boys but was more pronounced in girls from age 11 y, particularly among the more active girls (50^th^-97^th^ percentiles). Tables detailing percentiles values for each metric are presented in Additional file 10 (Additional file 9 for the equivalent tables relating to the MAD-derived metrics).

### Results for study aim 2

**Fig. 6:**
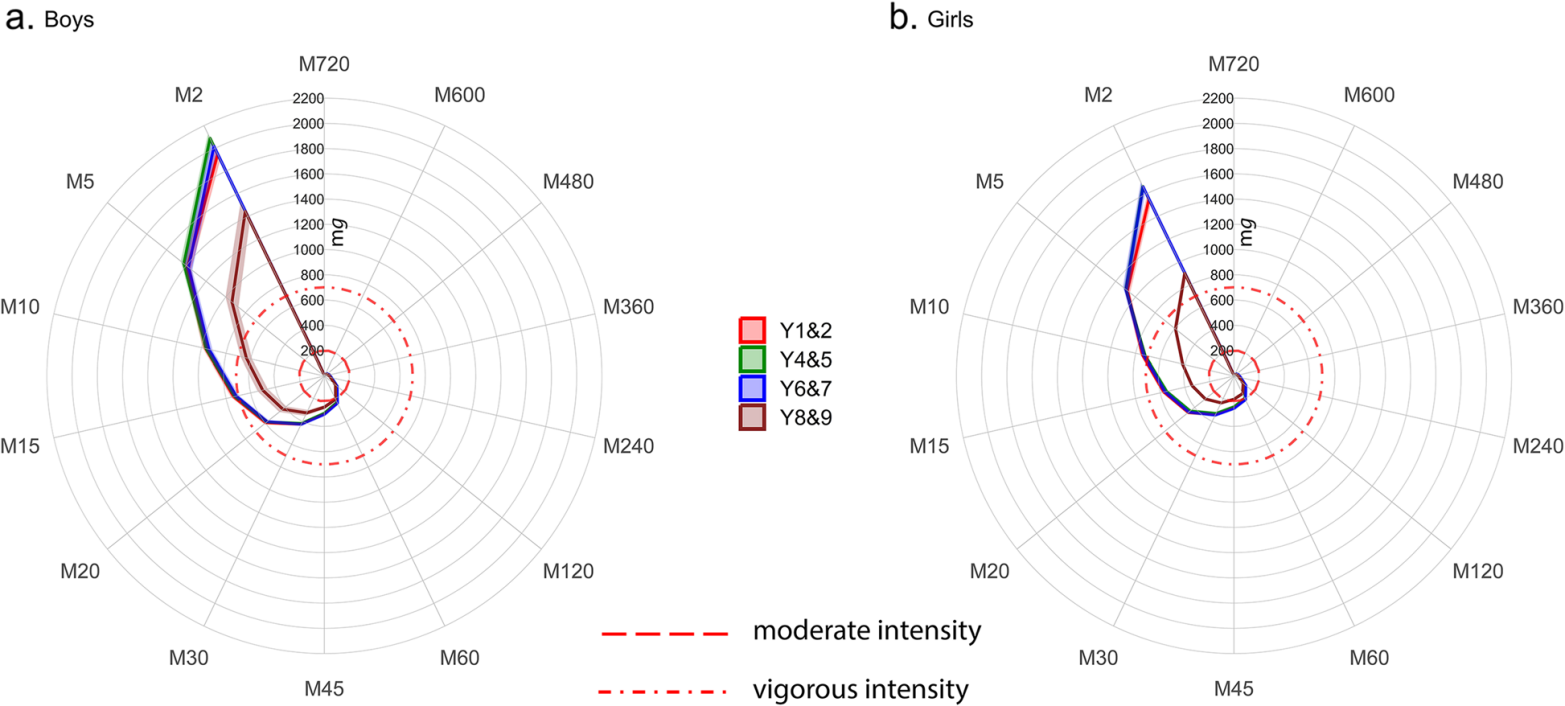
Waking hours physical activity profiles described by MX metrics for boys (panel **a**) and girls (panel **b**)

For each metric boys were more active than girls (*p* < 0.001) at all Year-groups (Table 3 and Additional file 11). For all metrics physical activity was lowest in the Y8&9 group, who were significantly less active than the Y1&2 group for average acceleration (*p* < 0.001), intensity gradient (*p* < 0.001), M2 to M60, and M360 metrics (*p* < 0.0001 to *p* = 0.048), and MVPA (*p* = 0.01). Figure 6 presents the range of MX metrics across age-groups for boys and girls, respectively, and shows that the main age-related differences in physical activity occurred among Y8&9 boys and girls. The plots illustrate the physical activity profiles underlying the statistical analyses, demonstrating how these differences were most apparent at higher intensities represented by the shorter duration MX metrics. This was more noticeable in girls and reflected the timing of the steepest age-related decline in intensity gradient. Using the indicative thresholds for MPA and VPA (dotted and dashed lines on the plots) [58], boys at all ages accumulated 60 min·d^−1^ in MVPA. Among girls 60 min·d^−1^ of MVPA was accrued by the Y6&7 group, with 45 min·d^−1^ achieved by the younger age groups, and around 30 min·d^−1^ by the oldest girls. Approximately 10 min·d^−1^ and 2 min·d^−1^ of VPA was achieved by the Y8&9 boys and girls, respectively. In contrast, the younger age groups accumulated VPA for 15 min·d^−1^ (boys) and 10 min·d^−1^ (girls).

The percentile curves and statistical analyses results by age-group and sex were similar for MAD generated metrics (Additional files 3, 4, 5, 6, 7 and 8).

## Discussion

This study is the first to report reference percentile values for ENMO- and MAD-generated average acceleration, intensity gradient, M60, and MVPA across a wide age range of boys and girls, derived from wrist-worn accelerometers. Our study is timely given the growing use of raw acceleration data and in particular, the ENMO metric to report comparable device-agnostic metrics describing youth physical activity. Providing reference values for these metrics may help researchers distinguish how physical activity volume or intensity (or both) differ by age and sex and where participants are positioned in terms of their activity levels.

Irrespective of age and metric used (ENMO or MAD), boys were more active than girls for all physical activity outcomes. This concurs with our previous findings in children [32] but contrasts with recent population-referenced data from the US, where girls’ mean MIMS-units**‧**day^−1^ were higher than boys’ from age 6 to 19 y [1]. MIMS-units reflect total physical activity volume and of the metrics reported in our study, are most comparable to average acceleration. A recent comparison of ENMO, MAD and MIMS-units to ActiGraph counts showed that ENMO and MAD increase proportionately more at higher intensities than MIMS-units and counts [59]. As boys’ undertook more high intensity activity, this difference between the metrics at higher intensities would likely affect boys’ overall activity volume more so than girls’, perhaps contributing to this discrepancy between studies [59]. Variations in girls’ and boys’ accelerometer wear time in the NHANES data [1], the inherent differences in the signal processing algorithms used to determine MIMS-units and ENMO, and the different population groups also likely contributed to these dissimilar findings.

The largest magnitude of differences between boys and girls was for the metrics related most to higher intensity physical activity (i.e., intensity gradient, lower duration MX metrics). This suggests that time in higher intensity physical activity was the main driver for the overall sex-related differences. This is consistent with findings from cut-point studies reporting significant sex differences in MVPA and VPA [60, 61] and our recent intensity spectrum work [62] which showed that the largest sex differences in 5-to-15 y olds occurred at accelerations ≥ 700 mg, which are indicative of activities with an equivalent intensity at least to jogging [63]. Such differences may reflect multidimensional influences on boys’ and girls’ structured physical activity, such as sports. For example, more opportunities typically exist for boys than for girls to do a wider range of organised out-of-school activities [64, 65], boys have superior perceived [66, 67] and actual [68, 69] motor competence than girls, and stronger parental social support for physical activity and praise for boys to engage in active pursuits such as sports have been reported [70, 71].

Age-related differences were similar for boys and girls across the four metrics. Of these metrics average acceleration is the most comparable to the recently published MIMS-unit reference values for youth aged 5 and 15 y [1]. However, Belcher et al. showed that MIMS-units·d^−1^ peaked at age 6 y in both sexes [1], which was earlier than we observed in our sample for any metric. The age at which physical activity was highest varied between ages 6.5 y through to 10 y across the different metrics and both sexes. Generally, average acceleration peaked earlier than metrics which had an intensity component; (e.g., intensity gradient typically peaked 1-year later than average acceleration). This could relate to age-linked increases in motor skill proficiency which predispose relatively older children to take part in more structured and higher intensity physical activities [72]. The only other comparable study of youth accelerometer reference values reported total activity counts and MVPA from NHANES hip accelerometer counts data collected between 2003 to 2006 [26]. This also showed boys’ and girls’ physical activity from both metrics peaking at age 6 y, but differed from our findings where average acceleration and MVPA were highest between ages 6.5–10 y. These differences were most likely associated with disparities in accelerometer-related factors (i.e., signal processing algorithms used to determine outcome metrics, device placement, selected cut-points), and the characteristics of the respective samples.

We observed that the age of peak physical activity also differed by activity level. For less active participants all physical activity metrics were highest around 1 y earlier than for more active peers. This could have reflected the influence of developmental differences and environmental factors that differentially active children are exposed to. A combination of variations in motor competence proficiency [68], physical activity and sport opportunities [65], structure and intensity of these opportunities [73], and psychosocial correlates [71] may have underpinned the observed age-related differences. Moreover, we found that across all metrics the highest reference values for girls occurred at younger ages than for boys. This likely relates to differences in the timing and tempo of maturation between boys and girls of the same chronological age [74], with girls typically undergoing physical changes earlier than boys. Studies have shown that when boys and girls are compared by biological age, sex- and chronological age-related differences in total physical activity and MVPA are attenuated [74, 75]. Further, recent review evidence supports the notion that maturational timing is inversely associated with overall physical activity and sports participation [76]. This evidence though is inconsistent, due in part to differences in study methodologies (e.g., maturity indicator, device vs. self-report physical activity assessments) and adjustment for important confounding variables such as chronological age [76].

The MX metrics demonstrated that age-related activity differences occurred mainly in relation to the Y8&9 group, which concurs with the percentile reference data. Further, these differences were most apparent at higher intensities (i.e., lower duration MX metrics), which reflects the higher tempo and intermittent nature of physical activity in younger children [77]. Moreover, there is greater engagement in structured physical activity and sport among children compared to adolescents [65] as a consequence of various factors (e.g., greater activity/sport sampling among younger vs. increased specialisation among older groups [78], age-related drop-out from structured sport [79], and increased academic and social time demands among adolescents). When indicative thresholds for MPA and VPA were overlayed on the MX radar plots, sex-differences in the time accumulated in MVPA were observed, but more striking was the modest duration of accumulated VPA particularly among the oldest group (i.e., 10 and 2 min·day^−1^ for boys and girls, respectively). This stark age-related difference in higher intensity physical activity is concerning, particularly in light of the known associated physical health benefits of VPA [62, 80].

Strengths of the study include using 24-h data to establish waking and sleep times that were specific to age-groups and day-types, which allowed the summary physical activity metrics to reflect actual waking hours durations, rather than them being defined by an arbitrary value (e.g., 16 h reflecting 07.00 to 23.00) [62]. Although we used stringent wear time criteria there was a high level of compliance to wearing the wrist accelerometers, with data available from 83% of participants who had some recorded accelerometer outcome data, which exceeds the compliance level reported in Belcher’s NHANES sample [1]. Further, participants in our study averaged 5.1 to 6.6 d of wear and average wear time of 14.0 h·d^−1^ from an average 14.6 h·d^−1^ waking hours. Additionally, use of standardised accelerometer data processing decisions with non-proprietary raw acceleration data gathered from different accelerometers allowed data from a large number of studies to be pooled. This approach enables comparability between wrist-accelerometer studies and thus can advance assessment of youth physical activity in future.

Study limitations include use of cross-sectional data that were not nationally representative of English youth; for these reasons the reference values are not intended to be generalised beyond the sample population. There were also disproportionately more girls than boys in the sample and relatively fewer children in the youngest age group. This should be taken into account when comparing percentile values between age- and sex-groups, by considering the potential for values to be less representative if sample characteristics vary substantially to the reference group. Furthermore, participation rate data was not available for all included studies, thus there was a risk of sampling bias which could have influenced the results. Acceleration recording frequency was 100 Hz in eight of the pooled studies and 30 Hz in two. ENMO and MAD describe accelerations averaged over a given epoch, and thus the influence of recording frequency should be minimal, particularly when the same epoch duration is used, as was the case in our pooled sample. However, we acknowledge that the lower sampling frequency in two of the ten studies may have impacted on the resultant output [81]. This is the largest pooled youth dataset reporting average acceleration, intensity gradient, and MX metrics and our analyses provide important age- and sex-specific reference data. We hope that this will provide the impetus for international efforts to pool raw acceleration data on larger scales in order to produce nationally-representative reference values for each acceleration metric.

## Conclusions

This is the first study to present age- and sex-specific reference values for average acceleration, intensity gradient, MVPA, and MX metrics. Physical activity volume and intensity peaked between ages 6.5–10.5 y, and were significantly lower in the Y8&9 group and girls. Our findings demonstrate the feasibility and utility of generating percentile curves for data-driven acceleration metrics to aid health-related physical activity research and promotion. We provide these values as a first step towards more comprehensive internationally representative reference value and view them as a marker for researchers to use when interpreting values from their own samples. In future there is a need to generate country-specific wrist-acceleration population-referenced norms for average acceleration, intensity gradient, and MX metrics. This would enable better quality physical activity surveillance, monitoring, and health promotion through standardised comparisons of device-independent accelerometer metrics across populations and subgroups.

## Supplementary Information

**Additional file 1. **Example GGIR config file listing of arguments and parameters used in the GGIR accelerometer data processing.

**Additional file 2. **Example GGIR physical activity metric output variables.

**Additional file 3. **Unadjusted mean values for all metrics by age-group and sex for MAD-derived metrics.

**Additional file 4. **Average acceleration percentile plots for MAD metric.

**Additional file 5. **Intensity gradient percentile plots for MAD metric.

**Additional file 6. **M60 percentile plots for MAD metric.

**Additional file 7. **Linear mixed models results for MAD metric.

**Additional file 8. **Radar plots showing intensity profile by age and sex for MAD MX metrics.

**Additional file 9. **Tables of percentile values for MAD metric.

**Additional file 10. **Tables of percentile values for ENMO metric.

**Additional file 11. **Linear mixed models results for ENMO metric.

**Additional file 12. **STROBE checklist.

**Additional file 13. **Recruitment and sampling information.

**Additional file 14. **Participant characteristics by study.

## Data Availability

The dataset analysed during the current study is available in the Open Science Framework repository from https://osf.io/rb9tx

## Abbreviations

BMI: Body mass index
ENMO: Euclidian Norm Minus One
GAMLSS: Generalized Additive Models for Location Scale and Shape
MAD: Mean Amplitude Deviation
MIMS-units: Monitor Independent Movement Summary units
MPA: Moderate physical activity
MVPA: Moderate-to-vigorous physical activity
MX: Minimum acceleration for the most active accumulated period of time, where X = the period of time
VPA: Vigorous physical activity
