# Supplementary material for: Reference values for wrist-worn accelerometer physical activity metrics in England children and adolescents
Source: Int J Behav Nutr Phys Act. 2023 Mar 25;20:35. doi: 10.1186/s12966-023-01435-z (PMC10039565; doi:10.1186/s12966-023-01435-z)
Supplement: Supplementary file 3 — Additional file 3. Unadjusted mean values for all metrics by age-group and sex for MAD-derived metrics. [file 12966_2023_1435_MOESM3_ESM.docx]

Participants’ unadjusted waking hours average weighted week accelerometer data, grouped by sex and age (Mean (SD), unless stated otherwise)

|  | Boys | | | |  | Girls | | | |
| --- | --- | --- | --- | --- | --- | --- | --- | --- | --- |
|  | Y1&2 | Y4&5 | Y6&7 | Y8&9 |  | Y1&2 | Y4&5 | Y6&7 | Y8&9 |
| *n* | 82 | 201 | 163 | 64 |  | 95 | 253 | 155 | 237 |
| Valid days (n) | 5.1  (0.5) | 6.4  (0.7) | 6.2  (0.7) | 6.1  (0.6) |  | 5.1 (0.4) | 6.4 (0.7) | 6.3  (0.6) | 6.6  (0.7) |
| Wear (hr⋅d^-1^) | 13.6  (0.8) | 13.9 (0.8) | 14.3 (0.8) | 14.5 (0.8) |  | 13.6 (0.6) | 13.9 (0.8) | 14.4 (0.7) | 14.6 (0.8) |
| Average acceleration (m*g)* | 88.3 (19.0) | 84.7 (19.9) | 85.4 (23.5) | 73.1 (22.6) |  | 79.9 (14.1) | 76.4 (18.0) | 79.4 (17.6) | 59.4 (13.4) |
| Intensity gradient | -2.30 (0.09) | -2.30 (0.13) | -2.32 (0.13) | -2.44 (0.15) |  | -2.36 (0.09) | -2.38 (0.10) | -2.39 (0.13) | -2.58 (0.12) |
| M2 (m*g*) | 1203.5 (171.5) | 1252.3 (203.0) | 1237.8 (228.8) | 1018.8 (243.6) |  | 1073.2 (139.0) | 1109.1 (181.9) | 1125.7 (220.8) | 774.8 (191.0) |
| M5 (m*g*) | 980.8 (148.0) | 1005.0 (191.8) | 989.8 (217.2) | 778.5 (211.0) |  | 865.6 (128.0) | 869.1 (165.4) | 882.1 (199.4) | 590.6 (141.2) |
| M10 (m*g*) | 793.9 (127.3) | 796.1 (173.5) | 784.7 (196.2) | 604.1 (172.5) |  | 693.2 (112.5) | 673.5 (142.2) | 687.1 (163.9) | 464.3 (101.9) |
| M15 (m*g*) | 677.2 (115.1) | 669.1 (158.0) | 660.8 (176.3) | 511.8 (144.8 |  | 587.4 (98.3) | 561.6 (124.0) | 574.9 (135.8) | 400.1 (81.5) |
| M20 (m*g*) | 592.1 (106.7) | 580.1 (143.5) | 574.9 (158.3) | 452.2 (123.7) |  | 511.6 (85.3) | 487.2 (108.6) | 499.7 (113.7) | 359.2 (69.0) |
| M30 (m*g*) | 474.0 (91.8) | 463.0 (118.2) | 462.8 (128.7) | 376.1 (92.8) |  | 410.5 (64.9) | 393.9 (85.5) | 404.7 (84.9) | 306.8 (54.1) |
| M45 (m*g*) | 368.3 (73.5) | 361.8 (89.4) | 364.6 (96.7) | 310.6 (70.7) |  | 322.8 (47.6) | 315.2 (65.1) | 325.9 (62.8) | 258.9 (43.4) |
| M60 (m*g*) | 304.0 (60.3) | 300.3 (69.6) | 304.8 (77.4) | 268.1 (59.3) |  | 269.7 (38.4) | 266.9 (53.7) | 277.7 (51.4) | 226.2 (38.5) |
| M120 (m*g*) | 180.6 (37.2) | 178.8 (38.0) | 187.0 (48.1) | 170.3 (40.9) |  | 164.1 (25.2) | 166.0 (35.2) | 177.4 (34.4) | 146.9 (29.7) |
| M240 (m*g*) | 84.9 (20.3) | 83.1 (21.7) | 90.6 (28.8) | 80.6 (25.6) |  | 78.5 (16.2) | 79.9 (22.1) | 88.0 (22.5) | 69.0 (19.5) |
| M360 (m*g*) | 67.4 (16.9) | 62.1 (17.6) | 65.1 (22.5) | 54.3 (19.3) |  | 62.5 (14.2) | 60.1 (18.4) | 63.3 (18.2) | 45.8 (14.9) |
| M480 (m*g*) | 41.5 (11.4) | 40.7 (12.8) | 45.6 (16.7) | 39.3 (14.5) |  | 38.5 (10.4) | 39.4 (13.6) | 44.0 (13.9) | 32.7 (11.5) |
| M600 (m*g*) | 19.0 (6.0) | 19.3 (6.9) | 22.1 (8.6) | 18.9 (7.5) |  | 17.4 (5.8) | 18.3 (7.4) | 20.5 (7.3) | 15.3 (6.0) |
| M720 (m*g*) | 7.9 (2.6) | 8.6 (3.1) | 10.2  (3.9) | 9.2 (3.6) |  | 7.2 (2.5) | 8.0 (3.4) | 9.0  (3.3) | 7.4  (2.7) |

Notes. Accelerometer outcomes calculated using the MAD metric; MX metrics = minimum acceleration for the most active accumulated X minutes
