# Supplementary figures and images for: Reference values for wrist-worn accelerometer physical activity metrics in England children and adolescents
Source: Int J Behav Nutr Phys Act. 2023 Mar 25;20:35. doi: 10.1186/s12966-023-01435-z (PMC10039565; doi:10.1186/s12966-023-01435-z)

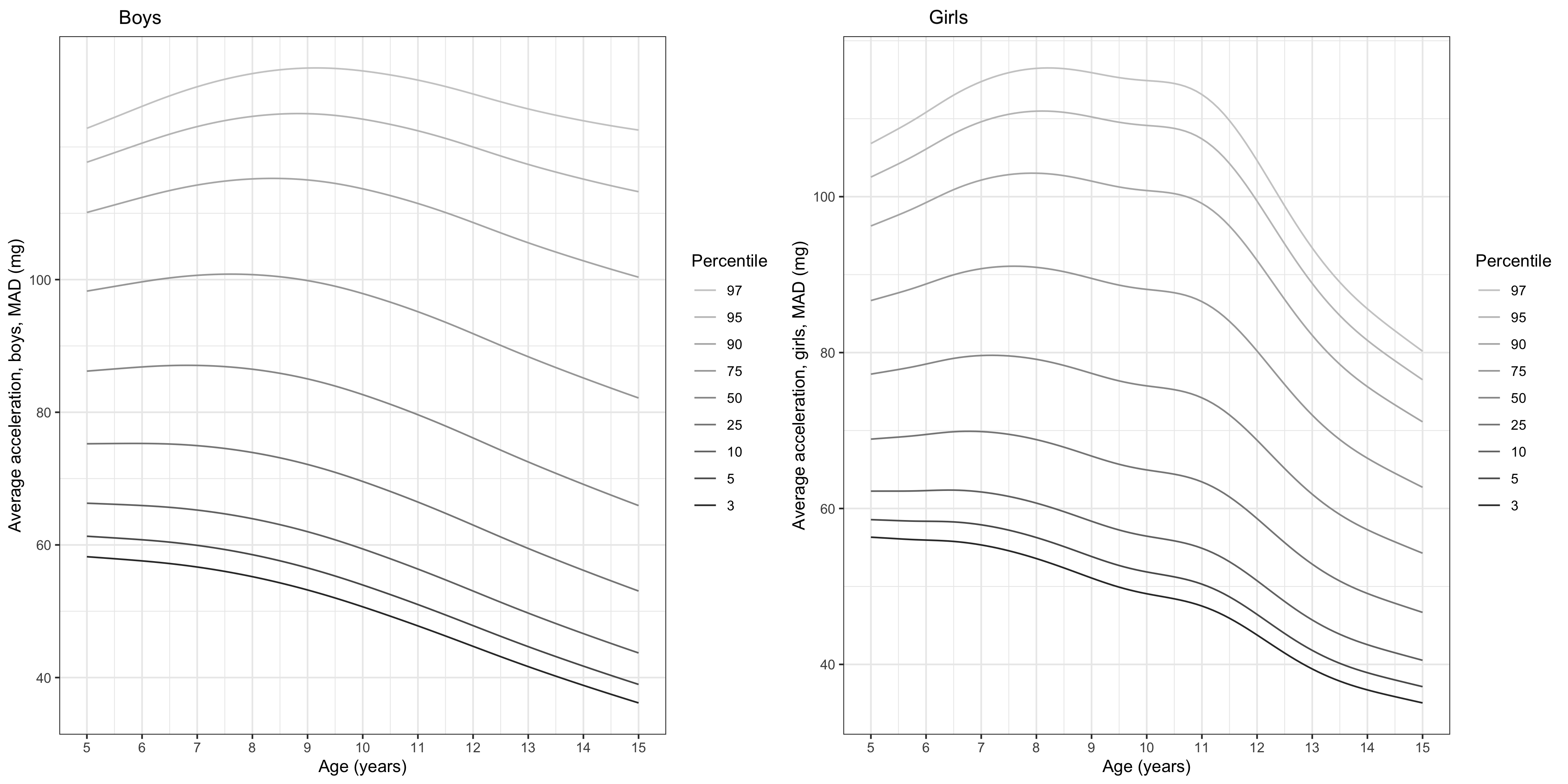

Supplement: Supplementary file 4 — Additional file 4. Average acceleration percentile plots for MAD metric. [file 12966_2023_1435_MOESM4_ESM.tiff]

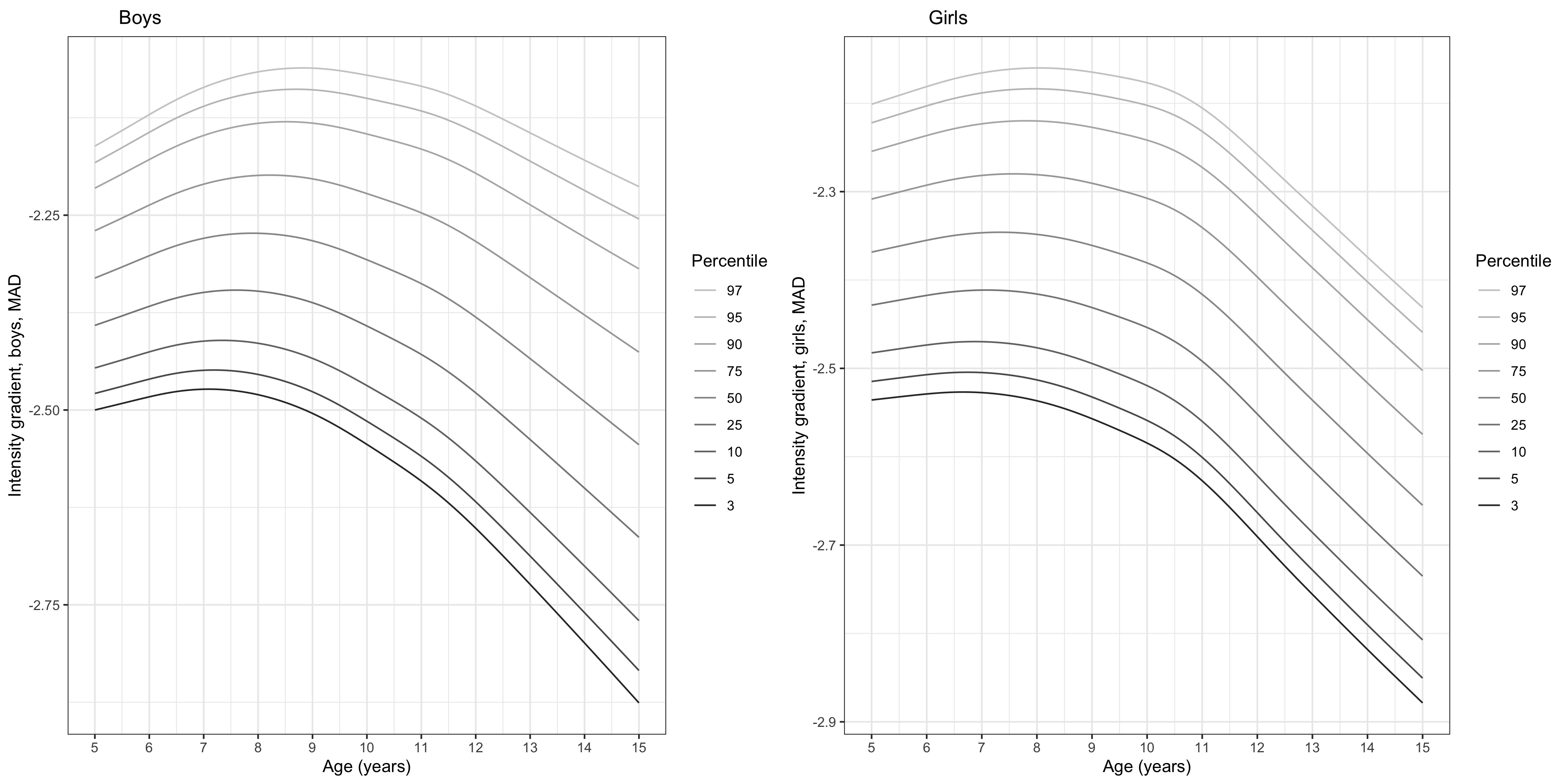

Supplement: Supplementary file 5 — Additional file 5. Intensity gradient percentile plots for MAD metric. [file 12966_2023_1435_MOESM5_ESM.tiff]

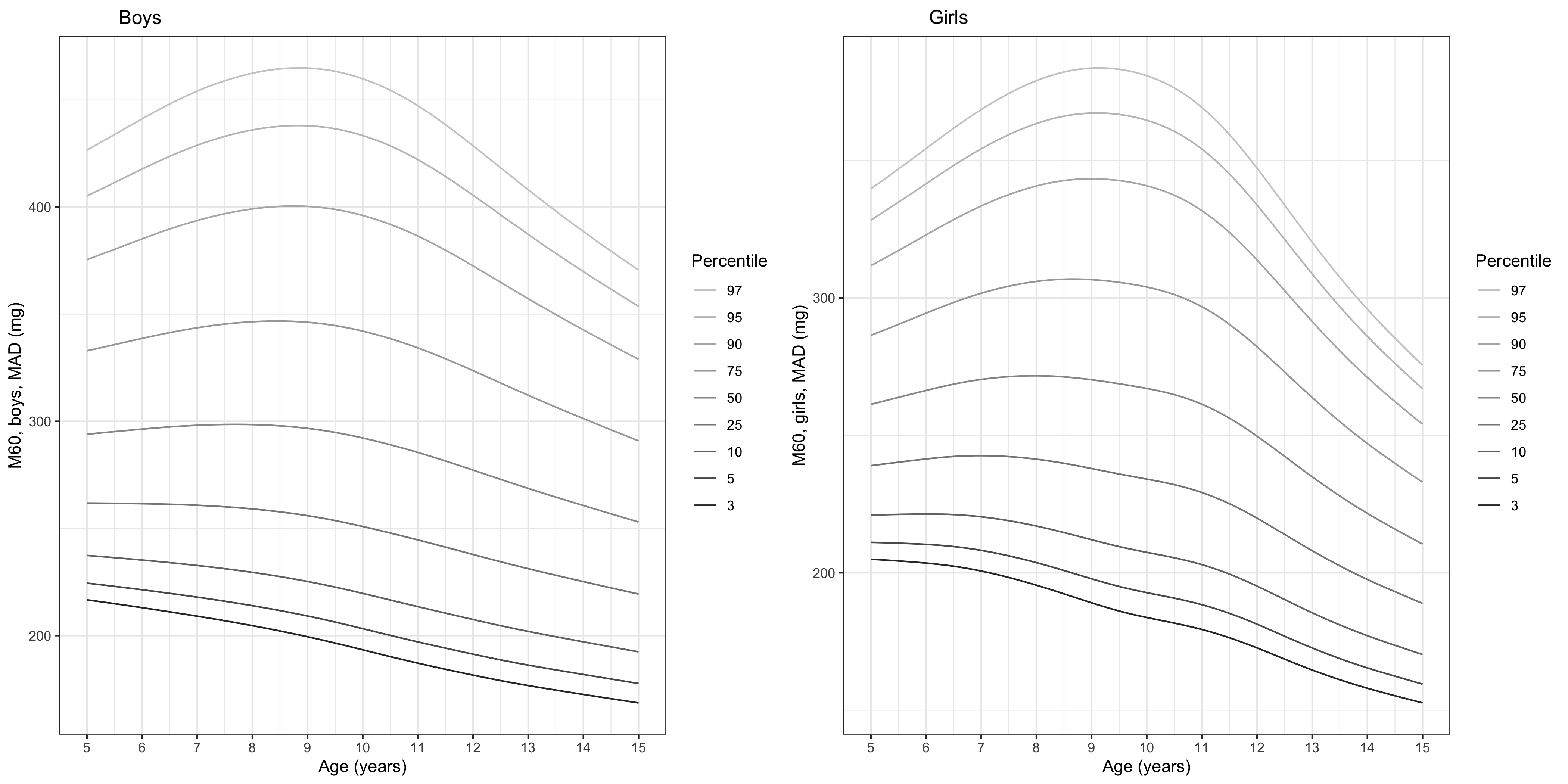

Supplement: Supplementary file 6 — Additional file 6. M60 percentile plots for MAD metric. [file 12966_2023_1435_MOESM6_ESM.tiff]
