# Supplementary material for: Reference values for wrist-worn accelerometer physical activity metrics in England children and adolescents
Source: Int J Behav Nutr Phys Act. 2023 Mar 25;20:35. doi: 10.1186/s12966-023-01435-z (PMC10039565; doi:10.1186/s12966-023-01435-z)
Supplement: Supplementary file 7 — Additional file 7. Linear mixed models results for MAD metric. [file 12966_2023_1435_MOESM7_ESM.docx]

Results of linear mixed model analyses of age and sex differences in MAD-derived metrics

| Average acceleration |  |  |  |  |
| --- | --- | --- | --- | --- |
| Grouping variable | ß | Lower 95% CI | Upper 95% CI | *p* |
| Sex† |  |  |  |  |
| Boys | 11.65 | 9.28 | 14.01 | <0.001 |
| Age group‡ |  |  |  |  |
| Y4&5 | -0.60 | -8.72 | 7.53 | 0.89 |
| Y6&7 | -5.02 | -12.76 | 2.72 | 0.21 |
| Y8&9 | -18.85 | -28.69 | -9.02 | <0.001 |
|  |  |  |  |  |
| Intensity gradient |  |  |  |  |
| Grouping variable | ß | Lower 95% CI | Upper 95% CI | *p* |
| Sex† |  |  |  |  |
| Boys | 0.08 | 0.06 | 0.09 | <0.001 |
| Age group‡ |  |  |  |  |
| Y4&5 | -0.01 | -0.07 | 0.04 | 0.58 |
| Y6&7 | -0.06 | -0.10 | -0.01 | 0.03 |
| Y8&9 | -0.19 | -0.26 | -0.13 | <0.001 |
|  |  |  |  |  |
| M2 |  |  |  |  |
| Grouping variable | ß | Lower 95% CI | Upper 95% CI | *p* |
| Sex† |  |  |  |  |
| Boys | 138.93 | 114.64 | 168.23 | <0.001 |
| Age group‡ |  |  |  |  |
| Y4&5 | 58.71 | -31.61 | 149.03 | 0.21 |
| Y6&7 | -11.09 | -98.00 | 75.82 | 0.80 |
| Y8&9 | -248.59 | -358.60 | -138.60 | <0.001 |
|  |  |  |  |  |
| M5 |  |  |  |  |
| Grouping variable | ß | Lower 95% CI | Upper 95% CI | *p* |
| Sex† |  |  |  |  |
| Boys | 125.61 | 104.09 | 147.13 | <0.001 |
| Age group‡ |  |  |  |  |
| Y4&5 | 29.25 | -52.43 | 110.93 | 0.49 |
| Y6&7 | -35.53 | -114.31 | 43.24 | 0.38 |
| Y8&9 | -240.88 | -340.49 | -141.26 | <0.001 |
|  |  |  |  |  |
| M10 |  |  |  |  |
| Grouping variable | ß | Lower 95% CI | Upper 95% CI | *p* |
| Sex† |  |  |  |  |
| Boys | 109.44 | 91.09 | 127.80 | <0.001 |
| Age group‡ |  |  |  |  |
| Y4&5 | 3.06 | -66.17 | 72.29 | 0.93 |
| Y6&7 | -49.59 | -116.31 | 17.13 | 0.15 |
| Y8&9 | -213.92 | 298.31 | -129.52 | <0.001 |
|  |  |  |  |  |
| M15 |  |  |  |  |
| Grouping variable | ß | Lower 95% CI | Upper 95% CI | *p* |
| Sex† |  |  |  |  |
| Boys | 95.54 | 79.46 | 111.62 | <0.001 |
| Age group‡ |  |  |  |  |
| Y4&5 | -6.21 | -66.01 | 53.59 | 0.84 |
| Y6&7 | -50.61 | -108.15 | 6.94 | 0.09 |
| Y8&9 | -180.51 | -253.34 | -107.68 | <0.001 |
|  |  |  |  |  |
| M20 |  |  |  |  |
| Grouping variable | ß | Lower 95% CI | Upper 95% CI | *p* |
| Sex† |  |  |  |  |
| Boys | 83.47 | 69.24 | 97.71 | <0.001 |
| Age group‡ |  |  |  |  |
| Y4&5 | -7.50 | -59.48 | 44.49 | 0.78 |
| Y6&7 | -45.41 | -95.34 | 4.51 | 0.08 |
| Y8&9 | -149.01 | -212.24 | -85.77 | <0.001 |
|  |  |  |  |  |
| M30 |  |  |  |  |
| Grouping variable | ß | Lower 95% CI | Upper 95% CI | *p* |
| Sex† |  |  |  |  |
| Boys | 63.88 | 52.48 | 75.29 | <0.001 |
| Age group‡ |  |  |  |  |
| Y4&5 | -2.78 | -43.22 | 37.66 | 0.89 |
| Y6&7 | -32.07 | -70.76 | 6.63 | 0.11 |
| Y8&9 | -101.37 | -150.45 | -52.28 | <0.001 |
|  |  |  |  |  |
| M45 |  |  |  |  |
| Grouping variable | ß | Lower 95% CI | Upper 95% CI | *p* |
| Sex† |  |  |  |  |
| Boys | 44.70 | 36.05 | 53.35 | <0.001 |
| Age group‡ |  |  |  |  |
| Y4&5 | 4.70 | -25.04 | 34.43 | 0.76 |
| Y6&7 | -16.84 | -45.18 | 11.50 | 0.25 |
| Y8&9 | -58.29 | -94.29 | -22.29 | 0.002 |
|  |  |  |  |  |
| M60 |  |  |  |  |
| Grouping variable | ß | Lower 95% CI | Upper 95% CI | *p* |
| Sex† |  |  |  |  |
| Boys | 33.21 | 26.22 | 40.20 | <0.001 |
| Age group‡ |  |  |  |  |
| Y4&5 | 8.89 | -14.93 | 32.72 | 0.47 |
| Y6&7 | -8.09 | -30.77 | 14.59 | 0.49 |
| Y8&9 | -35.28 | -64.10 | -6.46 | 0.02 |
|  |  |  |  |  |
| M120 |  |  |  |  |
| Grouping variable | ß | Lower 95% CI | Upper 95% CI | *p* |
| Sex† |  |  |  |  |
| Boys | 14.62 | 10.23 | 19.02 | <0.001 |
| Age group‡ |  |  |  |  |
| Y4&5 | 9.89 | -5.58 | 25.36 | 0.21 |
| Y6&7 | 1.66 | -13.12 | 16.45 | 0.83 |
| Y8&9 | -9.45 | -28.22 | 9.31 | 0.33 |
|  |  |  |  |  |
| M240 |  |  |  |  |
| Grouping variable | ß | Lower 95% CI | Upper 95% CI | *p* |
| Sex† |  |  |  |  |
| Boys | 5.43 | 2.73 | 8.13 | <0.001 |
| Age group‡ |  |  |  |  |
| Y4&5 | 6.08 | -3.65 | 15.80 | 0.23 |
| Y6&7 | 2.95 | -6.37 | 12.28 | 0.54 |
| Y8&9 | -3.89 | -15.70 | 7.93 | 0.52 |
|  |  |  |  |  |
| M360 |  |  |  |  |
| Grouping variable | ß | Lower 95% CI | Upper 95% CI | *p* |
| Sex† |  |  |  |  |
| Boys | 4.09 | 1.92 | 6.26 | <0.001 |
| Age group‡ |  |  |  |  |
| Y4&5 | 0.53 | -7.30 | 8.37 | 0.90 |
| Y6&7 | -4.26 | -11.77 | 3.26 | 0.27 |
| Y8&9 | -12.76 | -22.28 | 3.24 | 0.01 |
|  |  |  |  |  |
| M480 |  |  |  |  |
| Grouping variable | ß | Lower 95% CI | Upper 95% CI | *p* |
| Sex† |  |  |  |  |
| Boys | 3.03 | 1.40 | 4.65 | <0.001 |
| Age group‡ |  |  |  |  |
| Y4&5 | 2.69 | -3.04 | 8.42 | 0.36 |
| Y6&7 | 1.85 | -3.63 | 7.34 | 0.51 |
| Y8&9 | -3.04 | -10.00 | 3.92 | 0.40 |
|  |  |  |  |  |
| M600 |  |  |  |  |
| Grouping variable | ß | Lower 95% CI | Upper 95% CI | *p* |
| Sex† |  |  |  |  |
| Boys | 1.99 | 1.13 | 2.85 | <0.001 |
| Age group‡ |  |  |  |  |
| Y4&5 | 1.01 | -1.95 | 3.97 | 0.51 |
| Y6&7 | 1.23 | -1.59 | 4.05 | 0.40 |
| Y8&9 | -1.53 | -5.11 | 2.05 | 0.41 |
|  |  |  |  |  |
| M720 |  |  |  |  |
| Grouping variable | ß | Lower 95% CI | Upper 95% CI | *p* |
| Sex† |  |  |  |  |
| Boys | 1.06 | 0.68 | 1.44 | <0.001 |
| Age group‡ |  |  |  |  |
| Y4&5 | 0.52 | -0.72 | 1.77 | 0.41 |
| Y6&7 | 1.00 | -0.18 | 2.18 | 0.10 |
| Y8&9 | -0.02 | -1.52 | 1.48 | 0.98 |

Note. † girls were the reference group; ‡ Y1&2 were the reference group;

β values represent the difference in physical activity metric relative to the reference groups; all models adjusted for season, wear time, accelerometer model, and recording frequency
