# Supplementary material for: Reference values for wrist-worn accelerometer physical activity metrics in England children and adolescents
Source: Int J Behav Nutr Phys Act. 2023 Mar 25;20:35. doi: 10.1186/s12966-023-01435-z (PMC10039565; doi:10.1186/s12966-023-01435-z)
Supplement: Supplementary file 11 — Additional file 11. Linear mixed models results for ENMO metric. [file 12966_2023_1435_MOESM11_ESM.docx]

Results of linear mixed model analyses of age and sex differences in ENMO-derived metrics

| Average acceleration |  |  |  |  |
| --- | --- | --- | --- | --- |
| Grouping variable | ß | Lower 95% CI | Upper 95% CI | *p* |
| Sex† |  |  |  |  |
| Boys | 11.65 | 9.28 | 14.01 | <0.001 |
| Age group‡ |  |  |  |  |
| Y4&5 | -0.60 | -8.72 | 7.53 | 0.89 |
| Y6&7 | -5.02 | -12.76 | 2.72 | 0.21 |
| Y8&9 | -18.85 | -28.69 | -9.02 | <0.001 |
|  |  |  |  |  |
| Intensity gradient |  |  |  |  |
| Grouping variable | ß | Lower 95% CI | Upper 95% CI | *p* |
| Sex† |  |  |  |  |
| Boys | 0.10 | 0.09 | 0.12 | <0.001 |
| Age group‡ |  |  |  |  |
| Y4&5 | 0.01 | -0.04 | 0.07 | 0.65 |
| Y6&7 | -0.02 | -0.08 | 0.03 | 0.46 |
| Y8&9 | -0.19 | -0.26 | -0.12 | <0.001 |
|  |  |  |  |  |
| M2 |  |  |  |  |
| Grouping variable | ß | Lower 95% CI | Upper 95% CI | *p* |
| Sex† |  |  |  |  |
| Boys | 406.05 | 344.93 | 467.16 | <0.001 |
| Age group‡ |  |  |  |  |
| Y4&5 | 133.30 | -90.46 | 357.07 | 0.25 |
| Y6&7 | -4.07 | -219.02 | 210.87 | 0.97 |
| Y8&9 | -596.81 | -869.05 | -324.56 | <0.001 |
|  |  |  |  |  |
| M5 |  |  |  |  |
| Grouping variable | ß | Lower 95% CI | Upper 95% CI | *p* |
| Sex† |  |  |  |  |
| Boys | 305.39 | 258.64 | 352.14 | <0.001 |
| Age group‡ |  |  |  |  |
| Y4&5 | 33.86 | -134.80 | 202.52 | 0.70 |
| Y6&7 | -73.46 | -235.20 | 88.27 | 0.38 |
| Y8&9 | -485.48 | -690.46 | -280.49 | <0.001 |
|  |  |  |  |  |
| M10 |  |  |  |  |
| Grouping variable | ß | Lower 95% CI | Upper 95% CI | *p* |
| Sex† |  |  |  |  |
| Boys | 216.12 | 181.74 | 250.49 | <0.001 |
| Age group‡ |  |  |  |  |
| Y4&5 | -7.93 | -128.03 | 112.17 | 0.90 |
| Y6&7 | -79.05 | -193.76 | 35.66 | 0.18 |
| Y8&9 | -347.67 | -493.27 | -202.08 | <0.001 |
|  |  |  |  |  |
| M15 |  |  |  |  |
| Grouping variable | ß | Lower 95% CI | Upper 95% CI | *p* |
| Sex† |  |  |  |  |
| Boys | 163.91 | 136.07 | 191.75 | <0.001 |
| Age group‡ |  |  |  |  |
| Y4&5 | -11.94 | -103.46 | 79.58 | 0.80 |
| Y6&7 | -63.00 | -149.67 | 23.67 | 0.16 |
| Y8&9 | -251.39 | -361.71 | -141.07 | <0.001 |
|  |  |  |  |  |
| M20 |  |  |  |  |
| Grouping variable | ß | Lower 95% CI | Upper 95% CI | *p* |
| Sex† |  |  |  |  |
| Boys | §30.75 | 107.64 | 153.85 | <0.001 |
| Age group‡ |  |  |  |  |
| Y4&5 | -7.15 | -79.65 | 65.36 | 0.85 |
| Y6&7 | -46.24 | -114.40 | 21.92 | 0.19 |
| Y8&9 | -184.43 | -271.38 | 97.49 | <0.001 |
|  |  |  |  |  |
| M30 |  |  |  |  |
| Grouping variable | ß | Lower 95% CI | Upper 95% CI | *p* |
| Sex† |  |  |  |  |
| Boys | 86.02 | 68.96 | 103.09 | <0.001 |
| Age group‡ |  |  |  |  |
| Y4&5 | 2.14 | -48.45 | 52.73 | 0.93 |
| Y6&7 | -23.23 | -70.30 | 23.83 | 0.34 |
| Y8&9 | -107.09 | -167.30 | -46.87 | <0.001 |
|  |  |  |  |  |
| M45 |  |  |  |  |
| Grouping variable | ß | Lower 95% CI | Upper 95% CI | *p* |
| Sex† |  |  |  |  |
| Boys | 52.17 | 42.16 | 62.18 | <0.001 |
| Age group‡ |  |  |  |  |
| Y4&5 | 9.65 | -23.40 | 42.71 | 0.57 |
| Y6&7 | -7.81 | -39.14 | 23.51 | 0.63 |
| Y8&9 | -54.82 | -94.68 | -14.96 | 0.01 |
|  |  |  |  |  |
| M60 |  |  |  |  |
| Grouping variable | ß | Lower 95% CI | Upper 95% CI | *p* |
| Sex† |  |  |  |  |
| Boys | 36.20 | 28.68 | 43.71 | <0.001 |
| Age group‡ |  |  |  |  |
| Y4&5 | 11.28 | -13.33 | 35.89 | 0.37 |
| Y6&7 | -1.68 | -24.98 | 21.61 | 0.89 |
| Y8&9 | -30.52 | -60.17 | -0.86 | 0.048 |
|  |  |  |  |  |
| M120 |  |  |  |  |
| Grouping variable | ß | Lower 95% CI | Upper 95% CI | *p* |
| Sex† |  |  |  |  |
| Boys | 12.41 | 8.82 | 16.00 | <0.001 |
| Age group‡ |  |  |  |  |
| Y4&5 | 8.08 | -4.86 | 21.01 | 0.23 |
| Y6&7 | 2.47 | -9.93 | 14.88 | 0.70 |
| Y8&9 | -8.42 | -24.14 | 7.30 | 0.30 |
|  |  |  |  |  |
| M240 |  |  |  |  |
| Grouping variable | ß | Lower 95% CI | Upper 95% CI | *p* |
| Sex† |  |  |  |  |
| Boys | 4.28 | 2.48 | 6.08 | <0.001 |
| Age group‡ |  |  |  |  |
| Y4&5 | 3.15 | -3.33 | 9.63 | 0.34 |
| Y6&7 | 1.64 | -4.57 | 7.85 | 0.61 |
| Y8&9 | -3.58 | -11.45 | 4.29 | 0.38 |
|  |  |  |  |  |
| M360 |  |  |  |  |
| Grouping variable | ß | Lower 95% CI | Upper 95% CI | *p* |
| Sex† |  |  |  |  |
| Boys | 3.02 | 1.66 | 4.39 | <0.001 |
| Age group‡ |  |  |  |  |
| Y4&5 | -0.67 | -5.52 | 4.18 | 0.79 |
| Y6&7 | -3.06 | -7.70 | 1.59 | 0.20 |
| Y8&9 | -8.82 | -14.71 | -2.94 | 0.01 |
|  |  |  |  |  |
| M480 |  |  |  |  |
| Grouping variable | ß | Lower 95% CI | Upper 95% CI | *p* |
| Sex† |  |  |  |  |
| Boys | 1.96 | 0.98 | 2.93 | <0.001 |
| Age group‡ |  |  |  |  |
| Y4&5 | 0.45 | -2.92 | 3.81 | 0.80 |
| Y6&7 | 0.20 | -3.00 | 3.41 | 0.90 |
| Y8&9 | -3.16 | -7.24 | 0.91 | 0.13 |
|  |  |  |  |  |
| M600 |  |  |  |  |
| Grouping variable | ß | Lower 95% CI | Upper 95% CI | *p* |
| Sex† |  |  |  |  |
| Boys | 1.05 | 0.44 | 1.65 | <0.001 |
| Age group‡ |  |  |  |  |
| Y4&5 | -0.15 | -2.11 | 1.81 | 0.88 |
| Y6&7 | -0.15 | -2.00 | 1.70 | 0.88 |
| Y8&9 | -2.05 | -4.41 | 0.31 | 0.09 |
|  |  |  |  |  |
| M720 |  |  |  |  |
| Grouping variable | ß | Lower 95% CI | Upper 95% CI | *p* |
| Sex† |  |  |  |  |
| Boys | 0.55 | 0.15 | 0.94 | 0.01 |
| Age group‡ |  |  |  |  |
| Y4&5 | 0.52 | -0.83 | 1.87 | 0.46 |
| Y6&7 | 0.70 | -0.58 | 1.99 | 0.29 |
| Y8&9 | -0.06 | -1.69 | 1.57 | 0.94 |
|  |  |  |  |  |
| MVPA |  |  |  |  |
| Grouping variable | ß | Lower 95% CI | Upper 95% CI | *p* |
| Sex† |  |  |  |  |
| Boys | 10.98 | 8.40 | 13.57 | <0.001 |
| Age group‡ |  |  |  |  |
| Y4&5 | 1.95 | -7.00 | 10.91 | 0.67 |
| Y6&7 | -2.05 | -10.60 | 6.49 | 0.64 |
| Y8&9 | -15.38 | -26.23 | -4.53 | 0.01 |

Note. † girls were the reference group; ‡ Y1&2 were the reference group;

β values represent the difference in physical activity metric relative to the reference groups; all models adjusted for season, wear time, accelerometer model, and recording frequency
