# Supplementary material for: Reference values for wrist-worn accelerometer physical activity metrics in England children and adolescents
Source: Int J Behav Nutr Phys Act. 2023 Mar 25;20:35. doi: 10.1186/s12966-023-01435-z (PMC10039565; doi:10.1186/s12966-023-01435-z)
Supplement: Supplementary file 12 — Additional file 12. STROBE checklist. [file 12966_2023_1435_MOESM12_ESM.doc]

STROBE Statement—Checklist of items that should be included in reports of ***cross-sectional studies***

|  | Item No | Recommendation |
| --- | --- | --- |
| **Title and abstract** | 1 | (*a*) See title and abstract |
|  |  | (*b*) See abstract |
| Introduction | | |
| Background/rationale | 2 | See lines 81-137 |
| Objectives | 3 | See the lines 137-142 of the Background section |
| Methods | | |
| Study design | 4 | See Methods section ‘Data acquisition and study eligibility’ |
| Setting | 5 | See Methods section ‘Data acquisition and study eligibility’ |
| Participants | 6 | See Methods section ‘Data acquisition and study eligibility’ |
| Variables | 7 | See Methods section ‘Acceleration metrics’ |
| Data sources/ measurement | 8* | See Methods section ‘Acceleration metrics’ |
| Bias | 9 | Selection bias: The target group was primary and secondary school children and adolescents from northwest England. The participants were recruited from state schools in northwest England situated in a variety of urban and rural and socioeconomic locations. Participants were recruited by Year group and classes, and classes typically consisted of mixed ability students. Recruitment rates were high (typically ≥80%). For these reasons we are confident that the sample was representative of the target group and risk of selection bias was low.  Information bias: Accelerometer assessment protocols were standardised using accelerometers worn on the non-dominant wrist. Equivalence in raw acceleration output has been demonstrated between the two types of accelerometer used in the contributing studies. All raw accelerometer data were processed following a calibration procedure using the identical parameters and arguments in the same version of the GGIR R application. Accelerometer wear compliance was high based on stringent criterion of at least 3 valid weekdays and 1 valid weekend day of wear. Finally, the accelerometer outcomes are data driven so risk of misclassification into an incorrect physical activity intensity threshold was not present. Combined, we feel these procedures significantly minimise risk of measurement error. |
| Study size | 10 | Study size was arrived at by initial consideration of all eligible data from the contributing studies (n=2011), the removal of missing data (n=1503), and accelerometer data that did not meet the wear time criteria (n=1250) |
| Quantitative variables | 11 | See Methods section ‘Data analysis |
| Statistical methods | 12 | (*a*) See Methods section ‘Acceleration metrics’ |
|  |  | (*b*) See Methods section ‘Acceleration metrics’ |
|  |  | (*c*) Missing data were removed on a case-wise basis |
|  |  | (*d*) n/a |
|  |  | (*e*) n/a |
| Results | | |
| Participants | 13* | (a) See Figure 1 |
|  |  | (b) See Figure 1 |
|  |  | (c) See Figure 1 |
| Descriptive data | 14* | (a) See Table 2 and Results section, paragraph 1 |
|  |  | (b) See Figure 1 |
| Outcome data | 15* | See Table 3 |
| Main results | 16 | (*a*) See Figures 2-6 and Table 3 |
|  |  | (*b*) n/a |
|  |  | (*c*) n/a |
| Other analyses | 17 | n/a |
| Discussion | | |
| Key results | 18 | See Discussion section, paragraph 1 |
| Limitations | 19 | See Discussion section, lines 501-517 |
| Interpretation | 20 | See Conclusions section |
| Generalisability | 21 | See Discussion section, lines 501-503 |
| Other information | | |
| Funding | 22 | See Declarations section |

*Give information separately for exposed and unexposed groups.

**Note:** An Explanation and Elaboration article discusses each checklist item and gives methodological background and published examples of transparent reporting. The STROBE checklist is best used in conjunction with this article (freely available on the Web sites of PLoS Medicine at http://www.plosmedicine.org/, Annals of Internal Medicine at http://www.annals.org/, and Epidemiology at http://www.epidem.com/). Information on the STROBE Initiative is available at www.strobe-statement.org.
