# Supplementary material for: Reference values for wrist-worn accelerometer physical activity metrics in England children and adolescents
Source: Int J Behav Nutr Phys Act. 2023 Mar 25;20:35. doi: 10.1186/s12966-023-01435-z (PMC10039565; doi:10.1186/s12966-023-01435-z)
Supplement: Supplementary file 13 — Additional file 13. Recruitment and sampling information. [file 12966_2023_1435_MOESM13_ESM.docx]

**Additional sampling and recruitment information**

Sample recruitment

Ethically approved wrist accelerometry studies led or supervised by the first or last authors were identified for inclusion in this pooled individual participant data analysis. Eligible studies involved school-aged youth who had parental/carer written informed consent to participate in observational or intervention physical activity research studies during school term time. For inclusion in the analysis, studies required non-intervention assessments of wrist accelerometer-derived physical activity. For contributing intervention studies only baseline data were used. In addition to raw acceleration data, as a minimum, studies needed to provide stature, body mass, and demographic data including age and sex. Where published, details of these studies can be found elsewhere (1-6). Investigators with a major involvement in the eligible studies (e.g., past PhD students, co-supervisors) were approached by email and invited to contribute individual participant data to allow data harmonisation and subsequent pooled analysis. On receipt of signed data transfer agreements all contributing investigators transferred their de-identified data via a secure file sharing system. Data were available from 10 studies conducted in 71 schools between 2015 and 2022 in the Merseyside, Lancashire, and Greater Manchester counties of northwest England.

Representativeness of the sample

The target group was primary and secondary school children and adolescents from northwest England. The participants were recruited from state schools in northwest England situated in a variety of urban and rural and socioeconomic locations. Participants were recruited by Year group and classes, and classes typically consisted of mixed ability students. Recruitment rates were high (typically ≥80%). For these reasons we are confident that the sample was representative of the target group and risk of selection bias was low.

Comparisons between the analysed sample and the recruited sample

Our comparisons of demographic characteristics between the analysed and recruited samples showed that in general there were no differences between the group. The only exceptions were relatively more Y1&2 children in the analysed sample (4.8% difference) and relatively more Y4&5 children in the recruited sample (3.5% difference). Details of these comparisons are presented in the table below.

| Variable | Analytical sample | Recruited sample but excluded | *p* |
| --- | --- | --- | --- |
| Sex | Boys: 40.8%  Girls: 59.2% | Boys: 55.3%  Girls: 44.7% | 0.09 |
| Weight status | NW: 73.4%  OWOB: 26.6% | NW: 74.5%  OWOB: 25.5% | 0.60 |
| Age group | Y1&2: 14.1%  Y4&5: 36.2%  Y6&7: 25.8%  Y8&9: 23.9% | Y1&2: 18.9%  Y4&5: 32.7%  Y6&7: 25.7%  Y8&9: 22.7% | 0.03 |
| Age (yr) | 10.3 (2.4) | 10.1 (2.6) | 0.08 |
| BMI | 18.9 (3.8) | 18.6 (3.7) | 0.17 |
| BMI z-score | 0.52 (1.20) | 0.46 (1.26) | 0.29 |

Handling of missing data

From the 10 contributing studies n=1969 participants had informed parental consent to participate. Participants with missing descriptive data (n=76) were removed because we felt there would be too much uncertainty in the assumptions to replace height, weight, or BMI data. The 391 participants with missing accelerometer output data were removed because under a missing at random assumption there is no advantage in multiple imputation for missing data on outcome variables (7). The 312 participants who did not achieve the accelerometer minimum wear criteria were also removed because imputation of these summary activity outcomes would rely on too many unknown assumptions about the pattern of missingness and would thus introduce random variation.

Supported references

1. Hurter L, Fairclough SJ, Knowles ZR, Porcellato LA, Cooper-Ryan AM, Boddy LM. Establishing Raw Acceleration Thresholds to Classify Sedentary and Stationary Behaviour in Children. Children (Basel). 2018;5(12).

2. Crotti M, Foweather L, Rudd JR, Hurter L, Schwarz S, Boddy LM. Development of raw acceleration cut-points for wrist and hip accelerometers to assess sedentary behaviour and physical activity in 5-7-year-old children. J Sports Sci. 2020;38(9):1036-45.

3. Noonan RJ, Boddy LM, Kim Y, Knowles ZR, Fairclough SJ. Comparison of children’s free-living physical activity derived from wrist and hip raw accelerations during the segmented week. Journal of Sports Sciences. 2017;35(21):2067-72.

4. Owen MB, Kerner C, Taylor SL, Noonan RJ, Newson L, Kosteli MC, et al. The Feasibility of a Novel School Peer-Led Mentoring Model to Improve the Physical Activity Levels and Sedentary Time of Adolescent Girls: The Girls Peer Activity (G-PACT) Project. Children (Basel). 2018;5(6).

5. Taylor SL, Noonan RJ, Knowles ZR, Owen MB, McGrane B, Curry WB, et al. Evaluation of a Pilot School-Based Physical Activity Clustered Randomised Controlled Trial-Active Schools: Skelmersdale. Int J Environ Res Public Health. 2018;15(5).

6. Tyler R, Atkin AJ, Dainty JR, Dumuid D, Fairclough SJ. Cross-sectional associations between 24-hour activity behaviours and motor competence in youth: a compositional data analysis. Journal of Activity, Sedentary and Sleep Behaviors. 2022;1(1):3.

7. Sterne JAC, White IR, Carlin JB, Spratt M, Royston P, Kenward MG, et al. Multiple imputation for missing data in epidemiological and clinical research: potential and pitfalls. BMJ. 2009;338:b2393.
