# Supplementary material for: Reference values for wrist-worn accelerometer physical activity metrics in England children and adolescents
Source: Int J Behav Nutr Phys Act. 2023 Mar 25;20:35. doi: 10.1186/s12966-023-01435-z (PMC10039565; doi:10.1186/s12966-023-01435-z)
Supplement: Supplementary file 14 — Additional file 14. Participant characteristics by study. [file 12966_2023_1435_MOESM14_ESM.docx]

Participant characteristics for the included studies

| Study | Design | EIMD decile range | EIMD decile median (IQR) | School type | Sample n | Participation rate (%) |
| --- | --- | --- | --- | --- | --- | --- |
| 1 | Cross-sectional | 1-9 | 2 (1, 3) | Primary | 168 | 88 |
| 2 | Intervention | 1-9 | 2 (1, 2) | Primary & Secondary | 148 | 97 |
| 3 | Cross-sectional | 1-10 | 2 (1, 4) | Primary & Secondary | 103 | Data not available |
| 4 | Intervention | 1-10 | 7 (5, 9) | Secondary | 193 | 94 |
| 5 | Cross-sectional | 1-10 | 2 (2, 3) | Primary | 59 | 45 |
| 6 | Cross-sectional | 1-9 | 3 (2, 5) | Secondary | 29 | Data not available |
| 7 | Cross-sectional | 2-10 | 7 (6, 9) | Primary | 168 | Data not available |
| 8 | Cross-sectional | 1-7 | 1 (1, 2) | Primary | 95 | 40 |
| 9 | Intervention | 1-9 | 1 (1, 2) | Primary | 229 | 88 |
| 10 | Cross-sectional | 1-10 | 8 (6, 9) | Primary & Secondary | 311 | 55 |

Note. EIMD – English Indices of Multiple Deprivation
